# *Shank2* Mutant Mice Display Hyperactivity Insensitive to Methylphenidate and Reduced Flexibility in Social Motivation, but Normal Social Recognition

**DOI:** 10.3389/fnmol.2018.00365

**Published:** 2018-10-04

**Authors:** Elodie Ey, Nicolas Torquet, Fabrice de Chaumont, Julie Lévi-Strauss, Allain-Thibeault Ferhat, Anne-Marie Le Sourd, Tobias M. Boeckers, Thomas Bourgeron

**Affiliations:** ^1^CNRS UMR 3571, Human Genetics and Cognitive Functions, Institut Pasteur, Paris, France; ^2^Sorbonne Université, UPMC Univ Paris 06, INSERM, CNRS, Neuroscience Paris Seine – Institut de Biologie Paris Seine (NPS – IBPS), Paris, France; ^3^CNRS UMR 3691, BioImage Analysis, Institut Pasteur, Paris, France; ^4^Institute for Anatomy and Cell Biology, Ulm University, Ulm, Germany; ^5^Université Paris Diderot, Sorbonne Paris Cité, Human Genetics and Cognitive Functions, Paris, France

**Keywords:** mouse models, autism, *Shank2*, hyperactivity, methylphenidate, social motivation, ultrasonic vocalization, social recognition

## Abstract

Mouse models of autism can be used to study evolutionarily conserved mechanisms underlying behavioral abnormalities in social communication and repetitive behaviors. *SHANK* genes code for synaptic scaffolding proteins at excitatory synapses and mutations in all *SHANK* genes have been associated with autism. Here, we present three behavioral aspects of the mutant mice deleted for exon 16 in *Shank2*. First, we treated *Shank2* mutant mice with methylphenidate to rescue the hyperactivity. Our failure to do so suggests that the hyperactivity displayed by *Shank2* mutant mice is not related to the one displayed by the typical mouse models of hyperactivity, and might be more closely related to manic-like behaviors. Second, by testing the effect of group housing and social isolation on social interest, we highlighted that *Shank2* mutant mice lack the typical flexibility to modulate social interest, in comparison with wild-type littermates. Finally, we established a new protocol to test for social recognition in a social context. We used this protocol to show that *Shank2* mutant mice were able to discriminate familiar and unknown conspecifics in free interactions. Altogether, these studies shed some light on specific aspects of the behavioral defects displayed by the *Shank2* mouse model. Such information could be used to orient therapeutic strategies and to design more specific tests to characterize the complex behavior of mouse models of autism.

## Introduction

Autism spectrum conditions (ASCs; henceforth autism) are characterized by atypical social communication, including social interactions and verbal and non-verbal communication, as well as stereotyped and repeated behaviors and restricted interests (American Psychiatric Association, 2013). More than 80 genes have been robustly associated with autism (Abrahams et al., 2013). Among these genes, the *SHANK* family is of interest since mutations in each of the three members of this family (*SHANK1*, *PROSAP1/SHANK2*, and *PROSAP2/SHANK3*) have been identified in patients with autism, but with a gradient of severity (Leblond et al., 2014). Patients carrying a mutation in *SHANK1* display a mild phenotype in social communication and stereotypes, while patients carrying *SHANK2* mutations are more affected in their social interactions and repetitive behaviors. Patients with *SHANK3* mutations are even more severely affected and are also in the great majority of cases diagnosed with intellectual disability.

In the present study, we focused our experiments on mice lacking the Shank2 protein. To date, three different genetic constructions of the model exist (reviewed in Eltokhi et al., 2018): deletion of exon 16 [knock out (Schmeisser et al., 2012; Ey et al., 2013; Lim et al., 2016), conditional knock out in Purkinje cells (Peter et al., 2016)], deletion of exons 15 and 16 [knock out (Won et al., 2012; Lee et al., 2015; Lim et al., 2016), conditional knock out in Purkinje cells (Ha et al., 2016), conditional knock out in excitatory neurons (Kim et al., 2018), conditional knock out in inhibitory neurons (Kim et al., 2018), conditional knock out in parvalbumin-positive neurons (Lee et al., 2018)], deletion of exon 24 [knock out (Pappas et al., 2017), conditional knock out in Purkinje cells (Pappas et al., 2017), conditional knock out in excitatory neurons of neocortex and hippocampus (Pappas et al., 2017)]. All these models display hyperactivity, except the mice mutated conditionally only in Purkinje cells (Ha et al., 2016; Peter et al., 2016; Pappas et al., 2017). As proxies for the core symptoms of autism, a number of studies identified subtle abnormalities in the social domain [reduced interest for social interactions (Schmeisser et al., 2012; Won et al., 2012; Lee et al., 2015; Peter et al., 2016; Kim et al., 2018)] [but not in Ha et al. (2016); Lim et al. (2016); Pappas et al. (2017); and Lee et al. (2018)], reduced interest for social novelty (Schmeisser et al., 2012; Lee et al., 2015; Peter et al., 2016) [but not in Won et al. (2012) and Kim et al. (2018)], atypical ultrasonic communication (Schmeisser et al., 2012; Won et al., 2012; Ey et al., 2013; Ha et al., 2016; Kim et al., 2018), and increased stereotyped behaviors (Ha et al., 2016; Peter et al., 2016; Kim et al., 2018; Lee et al., 2018) [but not in Lee et al. (2015); Pappas et al. (2017); and Kim et al. (2018)].

In this study, we aimed at modulating the behavioral phenotype of the *Shank2*^Δex16-/-^ [hereafter *Shank2* (MGI: 5435698; Schmeisser et al., 2012)] mice using pharmacological treatment or social isolation. For the pharmacological treatment, we used methylphenidate (commercially available for medical use under the name Ritalin^®^), a treatment for individuals diagnosed with attention-deficit/hyperactivity disorder (ADHD) (Stepanova et al., 2017). To test for social interest, we modulated the motivation to interact with another mouse by including a period of social isolation prior to the social interaction test. Finally, a review of the existing protocols for social recognition in mice (see **Supplementary Material** – Review of social recognition protocols) highlighted that the habituation-dishabituation protocol was ethologically relevant. We adapted this protocol by testing simultaneously the mutant mouse and the wild-type mouse to control rigorously the investigation of social recognition in *Shank2* mutant mice.

## Materials and Methods

### Modulation Through Pharmacological Treatment

We tested males (placebo: 10 *Shank2*^+/+^, 10 *Shank2*^+/-^, 10 *Shank2*^-/-^; treatment: 10 *Shank2*^+/+^, 10 *Shank2*^+/-^, and 10 *Shank2*^-/-^) and females (placebo: 9 *Shank2*^+/+^, 10 *Shank2*^+/-^, 10 *Shank2*^-/-^; treatment: 9 *Shank2*^+/+^, 10 *Shank2*^+/-^, 10 *Shank2*^-/-^) aged of 4–6 months. Female mice were housed in groups of 2–4 per cage, while males were single-housed because of high aggressiveness. *Shank2* mutant mice were initially described in Schmeisser et al. (2012), backcrossed for more than 10 generations on C57Bl/6J. We injected the animals with methylphenidate (MPH; 30 mg/kg; intra-peritoneal injection) or saline solution, 1 h before the test. Whether mice received saline or MPH was randomly chosen before starting the experiment. We tested the animals in the openfield. One hour after the injection, the animals were left to freely explore a round openfield (1 m of diameter) for 30 min (100 lux). We measured the distance traveled and compared it between wild-type, *Shank2*^+/-^ and *Shank2*^-/-^ mice treated with either saline solution or methylphenidate using non-parametric Wilcoxon-Mann-Whitney U-tests given the small sample size and the non-normal distribution of the data.

### Modulation Through Social Isolation

We tested 16 *Shank2*^+/+^ and 13 *Shank2*^-/-^ adult females of 4–6 months of age (Schmeisser et al., 2012). We did not use *Shank2*^+/-^ mice in the remaining parts of the paper, given the subtlety of social defects in this genotype (Schmeisser et al., 2012). *Shank2*^+/+^ and *Shank2*^-/-^ mice were tested twice in the occupant-new comer test (Ferhat et al., 2016). On the first time, they were group-housed (2–4 mice per cage) from weaning on. On the second time 3 weeks later, they were isolated for 3 days before the test; this last data had been presented in the original study (Schmeisser et al., 2012). In the occupant-new comer test, a female mouse (occupant) was placed in a test cage (Plexiglas, 50 cm × 25 cm × 30 cm, 100 lux, with fresh bedding) in a soundproof chamber for 30 min habituation. After this time, an unfamiliar group-housed C57Bl/6J adult female (new comer) was introduced. Social interactions were recorded continuously (high-resolution Sony XCD-SX90CR video camera). Ultrasonic vocalizations were recorded simultaneously with a condenser ultrasound microphone Polaroid/CMPA, the interface UltraSoundGate 416-200 and the software Avisoft-SASLab Pro Recorded from Avisoft Bioacoustics (sampling frequency: 300 kHz; FFT-length: 1024 points; 16-bit format). Ultrasonic vocalizations were recorded for the pair of mice tested (one C57Bl/6J new comer and one from the *Shank2* strain, either a wild-type one or a mutant one) since we cannot distinguish the identity of the caller in such a setting. Nevertheless, previous studies suggested that the contribution of the new comer is minor in comparison with that of the occupant (Hammerschmidt et al., 2012). We recorded manually the time spent in contact and the latency for the first contact. We also measured manually the latency for the first ultrasonic vocalizations and the total number of ultrasonic vocalizations emitted. We manually established the distribution of ultrasonic vocalizations among the following call types (see Ey et al., 2013):

- “short”: call duration was less than 5 ms;
- “simple”: call duration was longer than 5 ms and the frequency range was smaller than 6.25 kHz (flat) or call duration was longer than 5 ms and the frequency range was larger than 6.25 kHz and there was only one direction of frequency modulation (upward or downward);
- “complex”: frequency modulations in more than one direction and frequency range larger than 6.25 kHz (modulated), or inclusion of one or more additional frequency components (harmonic or non-linear phenomena, but no saturation) but no constraint on frequency range (complex);
- “unstructured”: no pure tone component, “noisy” calls;
- “frequency jumps” (fq jumps): presence of one or more jump(s) in frequency, without time gap between the consecutive elements; it can include noisy parts within the pure tone call or not.

We used unpaired non-parametric Wilcoxon tests to compare the latency for the first contact, the total time spent in contact, the latency for the first call and the call rate between genotypes within the social condition (either grouped or isolated). We used paired non-parametric Wilcoxon tests to compare the latency for the first contact, the total time spent in contact, the latency for the first call and the call rate between social conditions (grouped or isolated) within genotypes. We used Chi-squared (with a Bonferroni correction for multiple testing) tests to compare the proportions of calls within and between social conditions or genotypes.

### New Protocol for Social Recognition

We set up a new habituation-dishabituation test. After carefully reviewing the different protocols available to test social recognition, we found three protocols using free social interactions: two-trial social recognition, social discrimination, habituation-dishabituation (see **Supplementary Material** – Review of social recognition protocols). We adapted the habituation-dishabituation protocol by testing a pair of female mice (one *Shank2*^+/+^ mouse and one *Shank2*^-/-^ mouse) with a juvenile C57Bl/6J female mouse as the stimulus mouse. By testing paired mice, we collected data from the mutant and its control mouse in exactly the same conditions. We tested 12 pairs of one *Shank2*^+/+^ adult female and one *Shank2*^-/-^ adult female (6 months of age), paired together at least 1 week before testing. Juvenile females were housed in groups of four upon arrival at the animal facility (3 weeks of age) and until the end of the experiment. Mice were identified through RFID chips implanted under isoflurane anesthesia and after local sub-cutaneous injection of lidocaine (at 4 weeks of age for the juveniles and at weaning for the tested mice). On the day of testing, mice were habituated to the testing room 20 min before the test. We then introduced the pair of *Shank2* mice in a test cage (50 cm × 50 cm × 30 cm, fresh bedding). The pair of mice was left to explore freely the test cage for 20 min. After this habituation time, we introduced an unknown C57Bl/6J juvenile female mouse (5 weeks of age) and let the mice freely interact for 2 min. After this first encounter, we took out of the cage the juvenile mouse and left it alone in a housing cage for 5 min. We then re-introduced it in the cage for 2 min. We repeated these steps for four encounters with the same juvenile. On the fifth encounter, we introduced another unknown C57Bl/6J juvenile female mouse (5 weeks of age) from another housing cage. During each encounter, a tracking system, Live Mouse Tracker, allowed to individually follow each of the three mice and to automatically extract the time spent in contact with the juvenile for each tested mouse (de Chaumont et al., 2018). We used unpaired non-parametric Wilcoxon tests to compare the total time spent in contact.

We used paired non-parametric Wilcoxon tests to compare the total time spent in contact between *Shank2*^+/+^ and *Shank2*^-/-^ mice (within each pair) for each juvenile encounter. We used paired non-parametric Wilcoxon tests to compare the total time spent in contact within each genotype between the fourth encounter and the fifth one.

### Ethic Approval Statement

All experiments involving animals complied with the European ethical regulation, and were validated by the ethical committee CETEA n°89, Institut Pasteur, Paris. These procedures were realized within the project APAFIS#7706-20 161 12317252460 v2.

## Results

### The Effects of Methylphenidate on Hyperactivity in *Shank2* Mutant Mice

**FIGURE 1:**
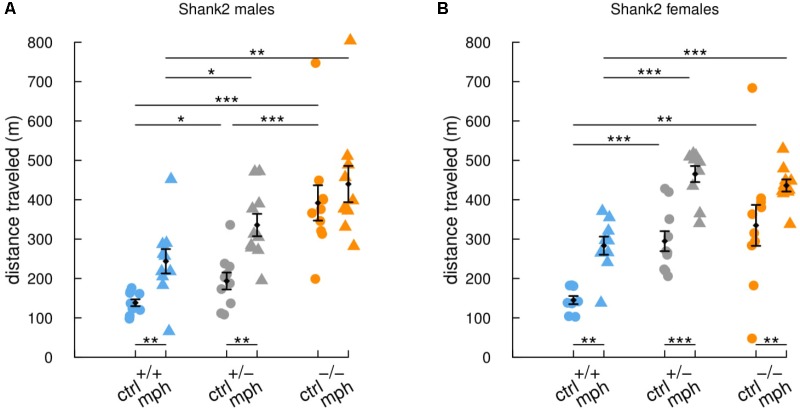
Similar enhancement of activity by methylphenidate treatment in *Shank2* wild-type and mutant mice. Distance traveled in 30-min free exploration of an openfield in **(A)** male and in **(B)** female *Shank2*^+/+^ (blue), *Shank2*^+/-^ (gray) and *Shank2*^-/-^ (orange) mice after methylphenidate treatment (MPH, 30 mg/kg) or after saline injection 1 h before the test. Data are presented as mean ± SEM, with individual points. Uncorrected non-parametric Wilcoxon tests were used (^∗^*p* < 0.05, ^∗∗^*p* < 0.01, ^∗∗∗^*p* < 0.001).

*Shank2^-^*^/^*^-^* mice are highly hyperactive in comparison with their wild-type littermates (Schmeisser et al., 2012). Here, we injected adult males and females with methylphenidate (MPH) or saline solution, 1 h before the openfield test. The saline-injected *Shank2^-^*^/^*^-^* mice traveled significantly longer distances in comparison with saline-injected *Shank2*^+/+^ mice (males: *W* = 0, *p* < 0.001; females: *W* = 10.5, *p* = 0.005) and *Shank2*^+/-^ mice (males: *W* = 7, *p* < 0.001; females: *W* = 0, *p* < 0.001; **Figure 1**). In addition, *Shank2*^+/-^ mice also displayed hyperactivity but to a lower extend in comparison with their wild-type littermates (males: *W* = 21.5, *p* = 0.034; females: *W* = 0, *p* < 0.001). These results confirmed the initially described gene dosage-related hyperactivity in this model (Schmeisser et al., 2012). MPH treatment increased significantly the distance traveled by *Shank2*^+/+^ mice (males: *W* = 10, *p* = 0.003; females: *W* = 4.5, *p* = 0.002) and by *Shank2*^+/-^ mice (males: *W* = 10, *p* = 0.002; females: *W* = 5, *p* < 0.001) in comparison with saline-injected mice of the same genotypes in both sexes. The MPH treatment also increased significantly the distance traveled by *Shank2^-^*^/^*^-^* female mice in comparison with saline-injected *Shank2^-^*^/^*^-^* female mice (*W* = 14, *p* = 0.005; **Figure 1B**). The difference in *Shank2^-^*^/^*^-^* male mice in comparison with saline-injected *Shank2^-^*^/^*^-^* males was not significant (*W* = 36, *p* = 0.307; **Figure 1A**), but the hyperactivity was already very high and we might have a saturation effect. In both sexes, MPH-injected *Shank2^-^*^/^*^-^* mice still traveled significantly longer distances in comparison with MPH-injected *Shank2*^+/+^ mice (males: *W* = 8, *p* = 0.002; females: *W* = 2, *p* < 0.001).

Overall, *Shank2*^-/-^, *Shank2*^+/-^, and *Shank2*^+/+^ mice reacted similarly to the injection of MPH, with an increase in their locomotor activity. MPH was therefore not efficient to rescue hyperactivity in this model.

### The Effects of Social Isolation on Social Behavior in *Shank2* Mutant Mice

Many mouse models of autism display only subtle abnormalities in classical behavioral tests for social interest (reviewed in Ferhat et al., 2017). One hypothesis is that the main social problem might reside in the social reward system, which can impair behavioral flexibility [see for instance the lack of social modulation of ultrasonic vocalizations in *Shank1* mutant mice (Wöhr et al., 2011)].

**FIGURE 2:**
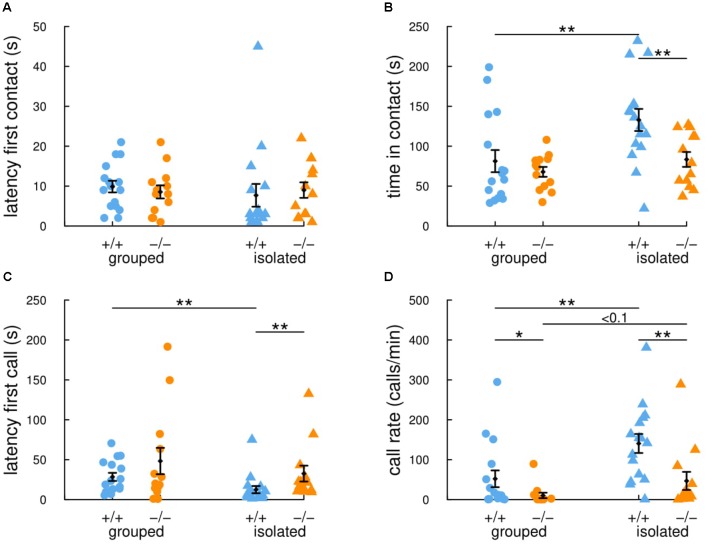
Previous social isolation modulates social communication in wild-type female mice but not in *Shank2* mutant female mice. In the 4-min (i.e., 240 s) occupant-new comer test, the latency to establish the first contact **(A)**, the total time spent in contact **(B)**, the latency to emit the first ultrasonic vocalization **(C)** and the number of calls per minute **(D)** were measured in group-housed mice and in mice isolated for 3 days before testing. Data are presented as mean ± SEM, with individual points. Uncorrected non-parametric Wilcoxon tests were used (^∗^*p* < 0.05, ^∗∗^*p* < 0.01, ^∗∗∗^*p* < 0.001).

**FIGURE 3:**
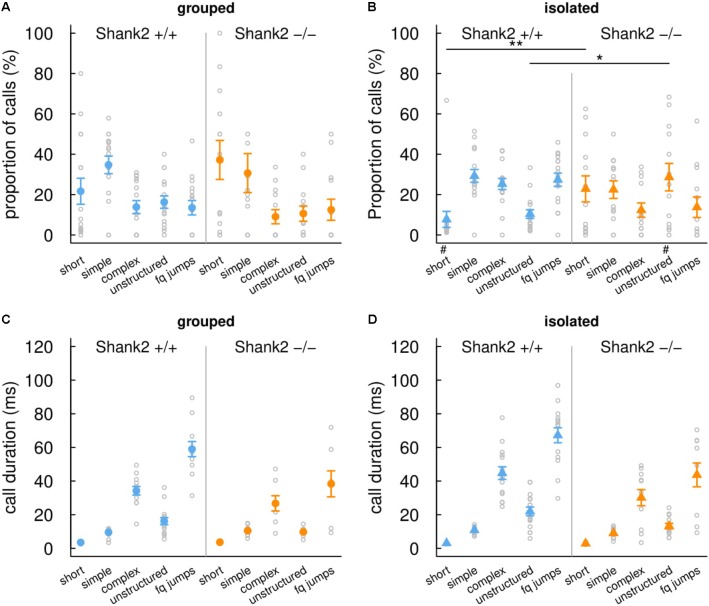
Effects of social isolation on the repertoire and duration of ultrasonic vocalizations emitted by *Shank2* females. Distribution of ultrasonic vocalizations among the five call types recorded during same-sex social interactions (4 min, i.e., 240 s) in group-housed females **(A)** and in females isolated for 3 days **(B)**. Mean duration of the five call types recorded during same-sex social interactions in group-housed females **(C)** and in females isolated for 3 days **(D)**. Data are presented as mean ± SEM, with individual points. Uncorrected Chi-squared tests were used to compare the proportion of each call type between pairs involving one wild-type and pairs involving one *Shank2* mutant mice within a social condition (^∗^*p* < 0.05, ^∗∗^*p* < 0.01) or between social condition (either grouped or isolated) within one genotype (^#^*p* < 0.05).

In this study, we therefore compared social interactions and ultrasonic vocalizations in the occupant-new comer test between group-housed and isolated adult female mice from the *Shank2* mutant strain. When females were group-housed, there were no significant differences between *Shank2*^+/+^ mice and *Shank2*^-/-^ mice in the latency for the first contact (*W* = 120.5, *p* = 0.482; **Figure 2A**), in the time spent in contact (*W* = 98.5, *p* = 0.826; **Figure 2B**), in the latency for the first call (*W* = 95, *p* = 0.714; **Figure 2C**), and in the vocal repertoire used (**Figures 3A,B**). The only difference was found in the number of vocalizations emitted, with less vocalizations emitted by *Shank2^-^*^/^*^-^* mice in comparison with wild-type mice (*W* = 150, *p* = 0.046; **Figure 2D**).

When females were isolated for 3 days, *Shank2^-^*^/^*^-^* mice spent significantly shorter time in contact with the new comer (*W* = 165, *p* = 0.008; **Figure 2B**) and emitted less ultrasonic vocalizations in comparison with *Shank2*^+/+^ mice (*W* = 171, *p* = 0.003; **Figure 2D**). Interestingly, when considering the ultrasonic vocalization repertoire used, *Shank2^-^*^/^*^-^* mice emitted significantly more calls from the short and unstructured categories than *Shank2*^+/+^ mice in the isolated condition (**Figures 3A,B**). In contrast, the duration of the calls for each call type did not differ significantly between social conditions or between genotypes (**Figures 3C,D**).

Overall, significant differences in social interaction and communication emerged between *Shank2*^-/-^ and *Shank2*^+/+^ mice only after social isolation, and not when animals were group-housed. These results suggested that *Shank2^-^*^/^*^-^* mice were impaired in their modulation of social motivation.

### Social Recognition in a Social Context in *Shank2* Mutant Mice

In their initial characterization, *Shank2*^-/-^ female mice displayed impairments in social recognition in the three-chambered test, with no significant preference for an unknown conspecific in comparison with a familiar conspecific (Schmeisser et al., 2012; Peter et al., 2016). However, in other studies using the *Shank2*^Δex15-16^ mice, social recognition in the three-chambered test did not differ significantly between homozygous mutant and wild-type (Won et al., 2012; Lee et al., 2015; Kim et al., 2018). In the present study, we aimed at testing whether social recognition was impaired when mice were freely interacting. We established a habituation-dishabituation test with a pair of adult female mice (one *Shank2*^+/+^ mouse and one *Shank2*^-/-^ mouse, familiar to each other for at least 1 week before the test) interacting repeatedly with a C57Bl/6J juvenile female. After four encounters with the same juvenile, the pair of mice encountered an unknown juvenile on the fifth encounter.

**FIGURE 4:**
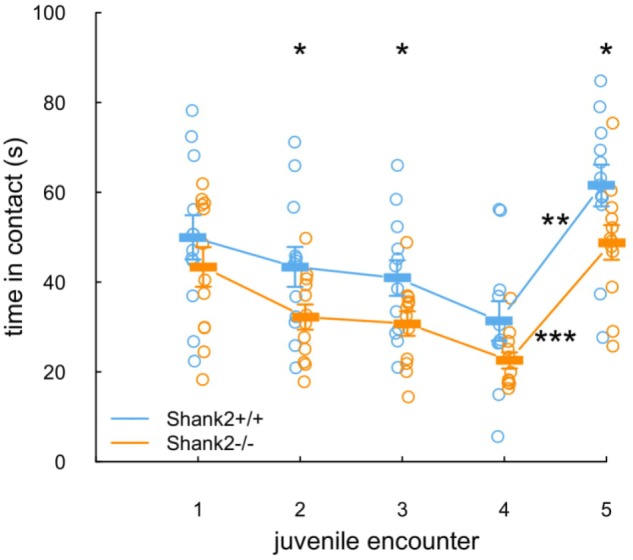
Unaffected social recognition in *Shank2* female mice. In the five repeated exposures (2 min, i.e., 120 s; inter-trial interval: 1 min, i.e., 60 s) of a pair of one *Shank2*^+/+^ mouse and one *Shank2*^-/-^ mouse to an unknown juvenile, the time spent in contact with the juvenile was measured for each tested mouse. The juvenile introduced was the same in encounters one to four; on encounter five, a new juvenile was introduced. Data are presented as mean ± SEM, with individual points. Uncorrected non-parametric paired Wilcoxon tests were used (^∗^*p* < 0.05, ^∗∗^*p* < 0.01, ^∗∗∗^*p* < 0.001).

The social recognition protocol appeared to be valid. Indeed, the time that *Shank2*^+/+^ mice spent in contact with the juvenile decreased over the four successive expositions to the same juvenile (**Figure 4**, blue line and dots). Then, they spent a significantly increased time in contact with the second juvenile presented for the fifth encounter (*W* = 1, *p* = 0.002), suggesting that they were able to discriminate their juvenile conspecifics. *Shank2^-^*^/^*^-^* mice followed a parallel profile despite the fact that they tended to spend shorter time in contact with the juveniles in comparison with their wild-type littermates (encounter 2: *W* = 65, *p* = 0.042; encounter 3: *W* = 66, *p* = 0.034; encounter 5: *W* = 65, *p* = 0.042; **Figure 4**, orange line and dots). As in *Shank2*^+/+^ mice, the time that *Shank2*^-/-^ mice spent in contact with the juvenile between the fourth and the fifth encounters also increased significantly (*W* = 0, *p* < 0.001).

Overall, the *Shank2*^-/-^ mice spent shorter time in contact with the juvenile in comparison with wild-type mice but remained able to differentiate two individuals.

## Discussion

### Methylphenidate Does Not Rescue the Hyperactivity of the Adult *Shank2^-^*^/^*^-^* Mice

The psychostimulant methylphenidate inhibits the reuptake of dopamine and norepinephrine (increase of dopamine and norepinephrine extracellular levels) (de la Peña et al., 2018). It is known to be an effective treatment for a subset of patients diagnosed with hyperactivity in humans (reviewed in Stepanova et al., 2017). It appeared to improve inattention, distractibility, hyperactivity and impulsivity more efficiently than placebo in patients with autism or neurodevelopmental disorders (Quintana et al., 1995; Handen et al., 2000; Research Units on Pediatric Psychopharmacology Autism Network, 2005; Kim et al., 2017). For instance, in a large study conducted by the Research Units on Pediatric Psychopharmacology Autism Network, about half of the patients with autism or neurodevelopmental disorders and hyperactivity responded to methylphenidate treatment (Research Units on Pediatric Psychopharmacology Autism Network, 2005). Nevertheless, methylphenidate appeared to be less efficient and to present more adverse effects (e.g., social withdrawal) in patients with autism than in children developing typical ADHD (Research Units on Pediatric Psychopharmacology Autism Network, 2005; Stepanova et al., 2017). Methylphenidate also tended to increase social withdrawal in patients with ADHD (Research Units on Pediatric Psychopharmacology Autism Network, 2005).

In mice, methylphenidate is used to test the predictive validity of mouse models for ADHD, since it can rescue the hyperactivity of several of these mouse models (de la Peña et al., 2018). In mouse models of autism, methylphenidate did not rescue hyperactivity in *Fmr1^-^*^/^*^-^* mice (Wrenn et al., 2015). In the first part of the present study, we highlighted that an injection of methylphenidate increased the activity of *Shank2*^+/+^ mice, as expected in wild-type mice, but also increased the activity of *Shank2*^+/-^ and *Shank2*^-/-^ mice. These results show that the hyperactivity of *Shank2^-^*^/^*^-^* adult mice is not attenuated by methylphenidate, and therefore might not be related to “ADHD-like hyperactivity.” Our results are consistent with those from Pappas et al. (2017) who also observed an increased hyperactivity in *Shank2*^Δex24^ mutant mice treated with amphetamine (2 mg/kg), while this hyperactivity was rescued by the mood stabilizers valproic acid or lithium (Pappas et al., 2017). These results suggest that the hyperactive phenotype in *Shank2* mutant mice could therefore be reminiscent of the manic-like phenotype of a mouse model for bipolar disorder. To further investigate this parallel with manic-like behavior, future studies on *Shank2* mouse models should include analyses on sleep-wake cycles, impulsivity and preference for reward stimuli (Beyer and Freund, 2017). Investigating the dopaminergic pathway in this model might also provide information on the relatedness of the phenotype of *Shank2* mice with a manic-like phenotype. With preliminary data in an eight-arm radial maze for spatial learning, we already noticed an increased arousal of *Shank2^-^*^/^*^-^* mice when seeking for the sweet reward in comparison with their wild-type littermates (unpublished data), suggesting an atypical reward seeking behavior. In parallel, clioquinol, a zinc chelator and a ionophore mobilizing trans-synaptic zinc has been shown to rescue the reduced social interest in the three-chambered test in mouse models of autism, but could not rescue the hyperactivity observed in the *Shank2*^Δex15-16^ model (Lee et al., 2015).

### Prior Isolation Increases Social Motivation for Conspecific, but Not in the *Shank2^-^*^/^*^-^* Mice

A reduction of social interactions might originate from abnormalities in the neuronal circuits involved in social reward. The strength of social interactions might then be modulated by the motivation of the individual to interact with peers. We aimed at testing whether mice lacking *Shank2* have the ability to modulate their interest in social interactions. We therefore manipulated their motivation to interact by housing them in groups or isolated.

While wild-type *Shank2*^+/+^ mice housed in isolation increased their social motivation with more time spent in contact and higher call rate compared to the group-housed condition, *Shank2*^-/-^ mutant mice behaved similarly, no matter whether in the isolated or group-housed conditions. Their level of behavioral flexibility in the social domain appeared to be lower in comparison with wild-type mice. Unfortunately, this mouse model has never been tested in a reversal learning task, to assess its cognitive flexibility. Other *Shank2* mouse models were tested for learning and memory but not in a reversal learning task, except the conditional, parvalbumin cell-type specific *Shank2*^Δex15-16^ knock-out mice that displayed typical learning and reversal learning in the Morris water maze task (Lee et al., 2018). Therefore, the cognitive flexibility of *Shank2* mutant mice remains to be further documented to understand whether the present lack of social flexibility is related to a reduced cognitive flexibility.

We could also hypothesize that *Shank2^-^*^/^*^-^* mice might consider social interactions as more aversive and therefore less rewarding than wild-type *Shank2*^+/+^ mice do. Indeed, our previous work showed that *Shank2*^-/-^ mice emit ultrasonic vocalizations with lower peak frequency in comparison with wild-type littermates (Ey et al., 2013). Interestingly, ultrasonic vocalizations emitted by adult males usually displayed a lower peak frequency in an aversive context (contention stress) than in affiliative social interactions (Chabout et al., 2012). Further experiments will focus on the modulation of the spontaneous home cage behavior between *Shank2*^-/-^ and *Shank2*^+/+^ mice to gather robust data on a more complete behavioral repertoire. Long-term group studies are now possible using a newly developed tracking system, Live Mouse Tracker (de Chaumont et al., 2018). The first experiments with mixed-genotype groups suggest that *Shank2^-^*^/^*^-^* female mice and their wild-type littermates differ in their involvement in complex social configurations (de Chaumont et al., 2018).

This reduced social reward could be in line with the hypothesis reviewed by Chevallier et al. (2012), in which social deficits in autism might come from social motivation deficits and therefore trigger social cognition deficits, and not the reverse. However, such an approach is still under discussion since evidences are mixed (reviewed in Bottini, 2018).

### *Shank2^-^*^/^*^-^* Mice Are Able to Integrate Social Cues for Recognition of Conspecific

The three-chamber test provides a binary way to measure the preference for social novelty versus social familiarity: it is a yes/no test (Crawley, 2004). It does not permit to disentangle whether the animal has a reduced motivation for (i.e., is not interested in) social novelty or whether it cannot distinguish the two different mice presented. The paradigm presented here tried to distinguish these two possibilities. We showed that *Shank2^-^*^/^*^-^* mice might be less motivated to interact with an unknown conspecific since they tend to spend shorter time in contact with comparison with their wild-type littermates. Nevertheless, they did not display social recognition impairments in this protocol: they still manage to gather information about the identity of the conspecific. *Shank2^-^*^/^*^-^* mice therefore are able to differentiate individuals from one another, i.e., to perceive and analyze social cues.

This is reminiscent of a series of experiments conducted in patients with autism. During social scene observation, toddlers with autism are less focused on eyes in comparison with neurotypical toddlers (Constantino et al., 2017), but they can be faster at identifying facial information compared with typically developed individuals (Gharib et al., 2015).

Whether the behavior of the *Shank2^-^*^/^*^-^* mouse is influenced by the behavior of its paired wild-type conspecific is unknown. If there were an influence of the behavior of the *Shank2*^+/+^ mouse on the *Shank2*^-/-^ mouse, this would mean that *Shank2*^-/-^ mice have the ability to socially imitate its paired conspecific, which depicts some interesting social competencies. If *Shank2*^-/-^ mice behave totally independently from the paired *Shank2*^+/+^ mice, this would mean that *Shank2*^-/-^ mice own social recognition ability. To disentangle these two possibilities, it would be interesting to try this protocol of social recognition with different types of pairings (*Shank2*^-/-^ vs. *Shank2*^-/-^, *Shank2*^-/-^ vs. *Shank2*^+/+^, and *Shank2*^+/+^ vs. *Shank2*^+/+^) and also with isolated mice.

## Conclusion

To summarize, the present study gathers new pieces of information on specific aspects of the behavior of the *Shank2^-^*^/^*^-^* mice. It shows that the hyperactivity displayed by this model cannot be reduced by methylphenidate. It also highlights that the social deficits displayed by this model might stem from a lack of flexibility in the social motivation. Finally, it provides more details on the social recognition deficits, which were not visible during direct social interactions with a conspecific, i.e., when the tested mice had access to all identity cues of the conspecifics. Interestingly, the reduced social interaction displayed by *Shank2^-^*^/^*^-^* mice might therefore be misinterpreted as a problem in social cognition. We propose an alternative hypothesis that *Shank2^-^*^/^*^-^* mice have high capacity to integrate social cues, but once these social cues are integrated, the mice display less social motivation to interact. Such information could be used to orient therapeutic strategies and to design more specific behavioral tests to characterize mouse models of autism.

## Data Availability Statement

The raw data supporting the conclusion of the manuscript will be made available by the authors, without undue reservation, to any qualified researcher.

## Author Contributions

EE, NT, A-TF, and JL-S performed and analyzed the experiments. A-MLS performed the genotyping of the mice. FdC created the tracking system for the social recognition experiments. TMB generated the mouse model. EE and TB conceived the project and wrote the manuscript.

## Conflict of Interest Statement

The authors declare that the research was conducted in the absence of any commercial or financial relationships that could be construed as a potential conflict of interest.
