# Supplementary material for: Shank2 Mutant Mice Display Hyperactivity Insensitive to Methylphenidate and Reduced Flexibility in Social Motivation, but Normal Social Recognition
Source: Front Mol Neurosci. 2018 Oct 4;11:365. doi: 10.3389/fnmol.2018.00365 (PMC6180161; doi:10.3389/fnmol.2018.00365)
Supplement: Supplementary file 1 [file Table_1.docx]

Supplementary material

**Review of social recognition protocols**

**Elodie Ey*, Nicolas Torquet, Fabrice de Chaumont, Julie Lévi-Strauss, Allain-Thibeault Ferhat, Anne-Marie Le Sourd, Tobias M. Boeckers, Thomas Bourgeron**

*** Correspondence:** Elodie Ey: elodie.ey@pasteur.fr

**1 Introduction**

Social recognition memory refers to the ability to separate familiar from unfamiliar conspecifics and to identify these items individually. Social recognition is a very rich and complex process, involving perception (through volatile and non-volatile cues (Brennan and Kendrick, 2006)), social motivation, recall, and discrimination abilities, by which a subject is aware that a stimulus has been previously experienced (Steckler et al., 1998). This innate ability to distinguish familiar from novel conspecifics is an essential feature of social behavior among group-living species, such as rodents. This skill allows the constitution of hierarchical social relationships among groups, and thus, is essential to the establishment of social bonds, such as pair bonds (Choleris et al., 2004; Johnson and Young, 2015; Winslow and Insel, 2004). It also enables an animal to assess the parasitic load of a conspecific and to avoid it as a social partner, hence decreasing the infection transmission risk (Kavaliers et al., 2004).

Since the 1980’s, many protocols have been published, aiming to assess and quantify social recognition among rodents. Despite their numerous differences, they are all organized in two sessions. During the first one, called the “learning session” or “sampling” (Engelmann et al., 2011a), the stimulus animal is presented to the experimental subject, either once or several times. This session is designed to allow the experimental subject to collect information on the stimulus subject. During the second session, the “test session”, the experimental subject is exposed, simultaneously or consecutively, to the stimulus animal presented during the learning session, and to a similar, novel animal, which had never met the experimental subject before. The two sessions are separated by a “retention interval”, ranging from a few minutes to a few days, during which the experimental animal is generally left alone in the experimental apparatus.

The objective of this review is to take stock of the different protocols used to assess social recognition in behavioral studies in mice. We would like to point out the most suited experimental conditions to test the discrimination abilities of the experimental mouse.

**Methods**

We searched in PubMed all the articles published between 1993 and 2013, as the first article studying social recognition among male mice was published in 1993 (Bluthé et al., 1993). We searched articles containing the words “social recognition” and “mice” in their title or abstract ((social recognition[Title/Abstract]) AND mice[Title/Abstract]), on August 2015. It led to 195 results. Searching all the articles containing “social discrimination” and “mice” in their title or abstract, but not “social recognition” (((social discrimination[Title/Abstract]) AND mice[Title/Abstract]) NOT social recognition[Title/Abstract]) led to 19 additional results. Searching all the articles containing “social memory” and “mice” in their title or abstract, but not “social recognition” or “social discrimination” ((((social memory[Title/Abstract]) AND mice[Title/Abstract]) NOT social recognition[Title/Abstract]) NOT social discrimination[Title/Abstract]) led to 53 additional articles. Among these 267 articles, we excluded some articles because they did not provide details on their experimental parameters, or because they did not exhibit any statistical analysis. We also chose to exclude from these 267 articles all the studies using the three-chamber test, or any test in which stimulus mice were physically constrained. Indeed, we considered that the whole olfactory signature of the stimulus mice has to be investigated by the experimental mouse. The use of physical constraints (enclosures, corrals, or compartments) could impair the sampling of the non-volatile fraction of the olfactory signature. Furthermore, it provides more detailed information about recognition because it allows the discrimination between different kinds of contacts: direct contact with the body surface, with the ano-genital region, or close following.

**Results**

Among the remaining articles (n=77) identified on PubMed, we found three different protocols used in free recognition studies (Choleris et al., 2009; Ferguson et al., 2000; Macbeth et al., 2009; Winslow and Insel, 2004): two-trial social recognition (a), social discrimination (b), and habituation – dishabituation (c). In the two-trial social recognition protocol (a), the experimental mouse is first exposed to a stimulus animal and, after a predetermined period of time, is either re-exposed to the same (familiar) animal, or presented to a novel stimulus animal. The social discrimination protocol (b) is very similar to the previous one, except that on the re-exposure trial, both the familiar and novel animals are presented simultaneously. Finally, in the habituation – dishabituation protocol (c), the experimental mouse is exposed several times (typically four) to the same stimulus mouse, and, in the final trial, it is then exposed to a novel unfamiliar animal.

The protocols (a) and (c) share the characteristic of comparing separately the investigation time of a novel and a familiar mouse, whether it be after a single presentation of the familiar mouse (a), or after repeated presentations (c). In contrast, the protocol (b) compares the investigation time during a simultaneous presentation of both the familiar and novel mouse, either after a single presentation of the familiar mouse (classic protocol), or after repeated presentations (see (Garelick et al., 2009; James et al., 2015; Kasahara et al., 2007; Sánchez-Andrade and Kendrick, 2011; Zhou et al., 2015)).

For convenience, we divided the studies in three categories: protocol two-trial social recognition (a): Table II (n=30 studies), protocol social discrimination (b): Table III (n=22 studies), and protocol habituation – dishabituation (c): Table IV (n=38 studies). The sum is equal to 90 and not 77 because eight articles used two different protocols in the same study (Bielsky et al., 2005; Ferguson et al., 2000; Hitti and Siegelbaum, 2014; Jacobs and Tsien, 2012, 2014; Lee et al., 2008; Marino et al., 2005; Wersinger et al., 2002), and five others (Garelick et al., 2009; James et al., 2015; Kasahara et al., 2007; Sánchez-Andrade and Kendrick, 2011; Zhou et al., 2015) used a mixed protocol, between (b) and (c), and thus are counted in both corresponding tables.

*Test cage and habituation to the testing environment*

**Table I:** Size of the test cage and mean ratio of the time spent investigating the stimulus animal over the total duration of the sampling phase.

|  | References | Total nb of studies | Mean ratio of investigation time over the total duration of sampling phase |
| --- | --- | --- | --- |
| < 250 cm² | (Bégou et al., 2008a) | 1 | 0.222 |
| 250-300 cm² | (Engelmann, 2009; Hädicke and Engelmann, 2013; Jüch et al., 2009; Wanisch et al., 2008) | 4 | 0.108 |
| 300-350cm² |  | 0 |  |
| 350-400cm² | (Ferguson et al., 2002; Noack et al., 2010; Zou et al., 2009) | 3 | 0.584 |
| 400-450cm² | (Kalkonde et al., 2011; Kogan et al., 2000; Zhou et al., 2015) | 3 | 0.471 |
| 450-500cm² | (Moretti, 2006) | 1 | 0.667 |
| 500-550cm² | (Gao et al., 2009; Ishikawa et al., 2014; Nomoto et al., 2012; Suzuki et al., 2011; Yang et al., 2013) | 5 | 0.411 |
| 550-600cm² |  | 0 |  |
| 600-650cm² | (Kasahara et al., 2007) | 1 | 0.333 |
| 650-700cm² | (Lukas et al., 2013) | 1 | NA |
| 1050-1100cm² | (Sánchez-Andrade and Kendrick, 2011) | 1 | NA |
| 1100-1150cm² | (Greco et al., 2013) | 1 | NA |
| 1400-1450cm² | (Rial et al., 2009a, 2009b; Xikota et al., 2008) | 3 | 0.378 |
| 4050-5000cm² | (James et al., 2015) | 1 | NA |

The size of the test cage did not seem to influence the mean percentage of investigation time during the sampling phase (Table I). No differences emerged in the number of studies testing the animals in their home cages (27/75), in a new mouse cage (23/75), or in a new cage different from those in which mice were housed (25/75). However, we observed that 12/25 studies from protocol (a) used a new mouse cage, 12/17 studies from protocol (b) used a new different cage, and 18/33 studies from protocol (c) tested their animals in their home cages. Habituation to the testing room was approximately 30 min in all studies, but habituation to the test cage might take more complicated patterns, the most complicated being repeated exposures over several consecutive days in a minority of studies.

Transferring experimental subjects into new cages just before or during the experiment could interfere with social recognition (Engelmann et al., 2011b). Thus, habituation time is a very relevant piece of information. For example, changing cages 10 min before the sampling session (or before the choice session) disrupts long-term recognition. Similarly, moving the experimental mouse after the sampling session to a new cage for 4 min and then returning it to its home cage to continue testing also had a distracting impact. But moving the experimental subject in its home cage to an adjacent room for 1-4 min immediately before the sampling session does not affect the recognition performance with an inter-trial interval (ITI) of 24 h (see also (Moura et al., 2011) for rats).

*Isolation of the tested mouse*

The question of whether mice have to be isolated before testing or not is debated. On the one hand, the first social recognition studies (1995-2005) displayed long isolation times (more than one week), in order to allow the experimental mouse to establish a home cage territory (Bégou et al., 2008b; Bielsky et al., 2004, 2005; Ferguson et al., 2001; Lee et al., 2008; Nelson et al., 2013; Satomoto et al., 2009; Wersinger et al., 2007) and maybe also to increase their social motivation. But we noticed in more recent studies a tendency to reduce isolation durations. Indeed, Kogan and colleagues showed that group-housed males displayed long-term recognition memory for 24 h and even 7 days, while singly-housed males only displayed 30-min social recognition memory (Kogan et al., 2000). This was confirmed in another study, where singly-housed males showed also only short-term recognition memory (tested with ITI = 1 h) (Bluthé et al., 1993). In addition, isolation has been reported to increase males’ aggressiveness towards stimulus mice, and in particular juvenile ones (Bluthé et al., 1993; Richter et al., 2005).

*Exposure time*

The exposure time is the time that the experimental mouse spends in contact with the stimulus mouse in the sampling and test sessions. This duration ranged from 45 s to 5 min, with a bias for each protocol. Indeed, protocols (a) mostly used 5 min trials (17/32), whereas protocols (b) mainly applied 4 min encounters (12/21), and protocols (c) used in majority 1 min trials (26/37). Moreover, we found protocols (b) for which the sampling session consisted of a habituation protocol (c) (four presentations of 1 min (Garelick et al., 2009), 2 min (James et al., 2015; Kasahara et al., 2007; Sánchez-Andrade and Kendrick, 2011), or 5 min (Zhou et al., 2015)). We also found protocols (c) with three 2 min trials of habituation, and then three 2 min trials of dishabituation with a novel mouse (Bluthé et al., 1993; Koliatsos et al., 2011). Last, we found one protocol (Faizi et al., 2012) adding a sixth trial after the dishabituation trial, during which the familiar mouse used for habituation was presented again. When comparing the mean percentage of investigation time of the stimulus during the first sampling session across every exposure times, we found that the longer the encounters were, the lower the percentages of investigation duration were.

*Inter-trial intervals*

The inter-trial interval ranged from 0 min to 7 days. During these inter-trial intervals, almost all studies let the experimental mouse in the test cage during this time. Indeed, manipulating the experimental mouse during and after the sampling session could interfere with long-term recognition memory in mice (Engelmann, 2009; Perna et al., 2015; Richter et al., 2005; Wanisch et al., 2008). Nevertheless, experimental mice were returned to their home cages when ITI = 24 h, while they were left in the test cage if ITI = 1 h (Jacobs and Tsien, 2012). In contrast, the stimulus mice were either kept individually in a small cage (Bluthé et al., 1993; Prediger et al., 2011; Rial et al., 2009b; Terranova et al., 1994; Xikota et al., 2008), or put back into their home cages (Kalkonde et al., 2011; Sinai et al., 2010). In general, water bottles were put back into their cages if ITI > 1 h. In some studies using the protocol (a), another control session with two different stimuli presented in each of the two trials was conducted just after (Ishikawa et al., 2014), 5 min after (Kalkonde et al., 2011), one day after (Bluthé et al., 1993; Kogan et al., 2000) or 3 days after (Ito et al., 2011) the test session. These supplementary expositions were done to check that the behaviors observed were really specific to social recognition.

*Characteristics of the tested mice*

The characteristics of the tested mice play a role in the test outcome. Using old (more than 12 months of age) experimental mice reduced the time of exploration (Terranova et al., 1994). In many cases, older animals performed less efficiently in the recognition test than younger ones (Garelick et al., 2009; Greco et al., 2013; James et al., 2015; Rial et al., 2009a; Sakamoto et al., 2011; Terranova et al., 1994, 1996). The protocol (c) might be more appropriate to test older animals than the two other protocols. In addition, reducing the inter-trial interval might help improve the performances of old animals.

The sexual status of the tested males is also of importance. Nine studies used sexually naïve mice (Bielsky et al., 2004, 2005; Hädicke and Engelmann, 2013; Imwalle et al., 2002; Jin et al., 2007; Kogan et al., 2000; McGowan et al., 2011; Prediger et al., 2011; Samuelsen and Meredith, 2011). Yet, according to Thor and Holloway (Thor et al., 1982), sexually naive male rats display longer exploration times when introduced to females or unfamiliar juveniles, but also greater aggressiveness. Their hypothesis is that the experience of sexual relations might enrich the animal with new sensory signals, which could allow it to be more efficient in social recognition. Even if this study has been conducted among rats, it may give clues for understanding mouse’s behavior. The tested and the stimulus mice were housed in groups of 2-5 animals in the majority of the studies (Bégou et al., 2008b; Bielsky et al., 2004, 2005; Bilkei-Gorzo et al., 2014; Bluthé et al., 1993; Cohen et al., 2002; Engelmann, 2009; Ferguson et al., 2002; Gao et al., 2009; Greco et al., 2013; Hädicke and Engelmann, 2013; Hitti and Siegelbaum, 2014; Ishikawa et al., 2014; Jacobs and Tsien, 2012; James et al., 2015; Jia et al., 2006; Jüch et al., 2009; Kasahara et al., 2007; Kercmar et al., 2011; Klemenhagen et al., 2013; Kogan et al., 2000; McGowan et al., 2011; Noack et al., 2010; Nomoto et al., 2012; Perna et al., 2015; Prediger et al., 2011; Rial et al., 2009a; Richter et al., 2005; Sakamoto et al., 2011; Sánchez-Andrade and Kendrick, 2011; Sinai et al., 2010; Suzuki et al., 2011; Terranova et al., 1996; Wanisch et al., 2008; Winslow and Camacho, 1995; Zou et al., 2009).

*Characteristics of the stimulus mice*

One necessary feature of the stimulus mice is that they must never have direct contact with the experimental mouse before the beginning of the experiment. The age and the gender of the stimulus mice varied between studies. Stimulus mice were either juvenile mice (25-40-day-old; 44/86) or adult female mice (34/86) or males (8/84, either sedated or not). The use of juvenile mice was often justified by the lower aggressiveness displayed by the experimental mouse toward younger mice (Kasahara et al., 2007; Macbeth et al., 2009; Pietropaolo et al., 2011; Thor and Holloway, 1981). Experimental male mice were also less aggressive toward females, but the phase of their estrous cycle could modify the male’s behavior; this is why ovariectomized females were often used. However, the protocol (c) could provide a solution to the aggressiveness issue, as the very short exposure time (1 min) does not seem to be long enough to observe fights or mounting behaviors (Klemenhagen et al., 2013). As replacement, some laboratories used 18-month-old female mice considered “sexually inactive” (Shiryaev et al., 2011; Vulih-Shultzman et al., 2007) or adult sedated males (Bégou et al., 2008b; James et al., 2015; Sánchez-Andrade and Kendrick, 2011).

Gender-matched juvenile mice were used in many studies (Engelmann, 2009; Ferguson et al., 2002; Hädicke and Engelmann, 2013; Jüch et al., 2009; Noack et al., 2010; Perna et al., 2015; Richter et al., 2005; Wanisch et al., 2008). However, two studies showed that the sex of the juvenile mice had no significant effect on the results of the social recognition test (Engelmann et al., 2011b; Noack et al., 2010). A study using different types of stimulus mice in the same protocol (c) did not report any significant difference in the test’s outcome or in the average investigation time, using female mice or juvenile male mice (Glynn et al., 2010). An additional study with protocol (c) used either ovariectomized females, or adult male mice as stimuli (Winslow and Camacho, 1995). They obtained a significant decrease during habituation for the ovariectomized females group, but a T1-T2-restricted decrease for the adult male group. Social recognition abilities were even found to be functional when facing cage mate (Winslow and Camacho, 1995).

To conclude, studies mainly used juvenile mice when applying the (a) or (b) protocol (23/29 and 12/19 respectively), and mostly ovariectomized females when using the (c) protocol (24/38). However, it remains unclear whether these conditions were chosen because of increased test efficacy or because of protocol transmission.

**Discussion**

Free interactions for social recognition tests are closer to ethological conditions in which mice use these abilities. However, it might present some drawbacks –that can be circumvented- compared to interactions in which one of the animals is physically constrained in enclosures. First, the experimental mouse could leave his own odor on the first stimulus during sampling, which could be a bias for social recognition during the test session. Nevertheless, this hypothesis was refuted (Engelmann, 2009; Engelmann et al., 2011b). Second, placing stimulus mice under enclosures allows to semi-automatize the analysis, which was not easy until recently for free interactions. But new tracking systems have been developed and allow individual mice tracking during free social interactions (de Chaumont et al., 2018). Third, the investigation time depends on the stimulus’ own motivation and stress. For example, a very curious stimulus mouse follows and solicits the experimental mouse, then triggering investigation behaviors from the later. Thus, free interaction protocols request testing more individuals, in order to lower the weight of the stimuli behaviors on the final results. Finally, male mice display aggressive behaviors, which could increase their stress and distract them from their sampling activities. Radical solutions (e.g., using a “volatile-fraction cage”, which allows to give off only the smell of the stimuli (Macbeth et al., 2009), anesthetizing the stimuli (Bégou et al., 2008b; James et al., 2015; Sánchez-Andrade and Kendrick, 2011)) are not compatible with free interactions. Other methods to reduce aggression in male laboratory mice include avoiding single housing, transferring nesting material when cleaning the cages or reducing the habituation time in the test cage from 2 h to 20 min (Van Loo et al., 2003). In addition, groups composed of three mice showed the least aggression rate as compared to groups of five or eight mice. Indeed, dominance hierarchy seems to be more stable in smaller groups (Van Loo et al., 2003). Last, to extinguish aggressiveness (toward males) or sexually-motivated behaviors (toward females), several studies organized training sessions before the beginning of the experiments, in which stimulus males(Jacobs and Tsien, 2012) or ovariectomized females (Ferguson et al., 2001; Lee et al., 2008; Prediger et al., 2011; Takayanagi et al., 2005; Wersinger et al., 2002, 2007; Xikota et al., 2008) were presented over repeated days to the experimental animals. To reduce anxiety, experimental mice may also be handled each day before the experiment, for several days (Gao et al., 2009; James et al., 2015; Koliatsos et al., 2011; Nomoto et al., 2012; Sinai et al., 2010; Suzuki et al., 2011). They could also be habituated to handling and transport from colony to test room three days before the experiments (Hitti and Siegelbaum, 2014).

Considering all the studies that we gathered, we can point out the advantages of the three protocols. The protocol (a) is the simplest procedure to conduct, but also the least rich one, since the behavioral traits that can be examined are limited to one presentation with one individual at each time. The protocol (b) has been shown to be a highly sensitive mean of assessing social recognition because it has allowed the emergence of social discrimination in animals that appeared to possess limited social recognition abilities when tested in the habituation/dishabituation paradigm (Engelmann et al., 1995). Moreover, in this protocol, animals can be tested repeatedly, over different conditions, then serving as their own control, which increases the statistical power (Engelmann et al., 1995). The protocol (c) provides a way to distinguish subtle differences in social recognition: for example a habituation to the stimuli that would occur only from the third presentation. Moreover, this way of assessing social recognition enables to mimic the animals’ natural social encounters (Schellinck et al., 1995). However, a drawback of the (c) protocol is the necessity of repeatedly testing the same animal. Indeed, in the dishabituation trial, the level of investigation should return to its value during the first trial. In most studies, there is only a significant increase between the last habituation trial and the dishabituation one, but the level of investigation during the dishabituation trial is often still significantly lower than the one during the first trial. This suggests a sensitization to the testing procedure (Engelmann et al., 1995).

To conclude, the social recognition test allows the assessment of a main feature in mouse’s social abilities: the capacity to recognize conspecifics. Measuring the lowering of the investigation time of a familiar stimulus mouse compared to an unfamiliar one is a very partial view of mice’s social interactions. Social behavior embraces many other capacities, such as dominance, pair bonding, mother-pups interactions, or gender recognition.

**Table II:** Summary of studies using the protocol two-trial social recognition (a). We gathered information on the main parameters varying across laboratories and protocols. We also included a column for each protocol’s outcome in standard conditions, noting down the parameters for which the researchers obtained a significant p-value of investigation time decrease. Whenever possible, we also included in the results column, as a comparison and standardization element among protocols, the fraction of time spent by the experimental mouse exploring the first stimulus mouse in the first trial (R). Indeed, it is the only data that is always measured across all protocols. Abbreviations: LDC: Light-Dark Cycle; RLDC: Reversed Light-Dark Cycle; F: Food; W: Water; ad lib: ad libitum; EM: Experimental Mouse; SM: Stimulus Mouse; TC: Test Cage; H: Habituation; I=Isolation; TR: Test Room; MC: Mouse Cage; HC: Housing Cage; ITI: Inter-Trial Interval.

|  | **Strain** | **Experimental mouse’s features** | **Stimulus mice’s features** | **Environmental conditions** | **Housing conditions** | **Testing room** | **Isolation habituation** | **Control with a novel stimulus** | **Exposure time** | **Inter-trial interval** | **Number of individuals** | **Results (comparison of exploration time between familiar and unfamiliar conspecific in the second trial and ratio of exploration time over the total exposure duration in the first trial)** |
| --- | --- | --- | --- | --- | --- | --- | --- | --- | --- | --- | --- | --- |
| (Bluthé et al., 1993) | DBA/2 | -Age : 8 - 11 weeks | -Age : 21-35 days  -Males | -12 : 12 LDC  -L.on : 4pm  -dark phase  -F&W ad lib | -EM group-housed (3) -SM group-housed (10) -546cm²  -wait 2 mth | -Red light | I = 1 to 6days | yes | Max 4min, criterion of neglect (20s) | 20, 60, 120, 180 min | N=11 | ITI = 20 or 60 min : p<0.001 ITI = 120 or 180min : failure 0.5 – 20min : R=0.167 / 60min : R=0.25 |
| (Terranova et al., 1994) | NMRI | -Age : adults (2-3 month-old), aged (12-14 month-old), | -Age : 3 week-old  -males | -12:12 RLDC  -L.on : 7 pm  -dark phase  -T: 19-23°C  -F&W ad lib | ? | -IR light | -SM : I=30min  -test in HC | no | 5min | 5,30,60min | N=8-10 | No differences in investigation time between old/adult: R=0.333. Significant results for adult only, for ITI= 5min and 30min |
| (Terranova et al., 1996) | EM : CD1 | -Age : 3-4 month-old OR 11-13 month-old | -Age : 3 week-old  -males | -12: 12 RLDC  -L.on : 7 pm  -dark phase  -T : 19-23°C  -F&W ad lib | -EM group-housed (10)  -SM group-housed (5) | -Red light | EM: I=3 weeks in HC  SM: I=30min | no | 5min | 30min | N=7-10 | Significant difference for the 3-4 month-old group only.  R=0.45 |
| (Kogan et al., 2000) | C57BL/6J | -Age : 4-8 months  -Naives | -Age : < 5weeks  -Males | -12:12 LDC  -light phase -F&W ad lib except during encounters | -EM group-housed (2-5)  -432cm² | -dim light | H=30min MC | yes | 2min | 30min, 1, 3, 7 days | N=12 | (On 2min) 1: 60s, F: 40s –2 : 50s, N : 50s p<0,001 (for the decrease in the explo time of the familiar) R=0.5 – 0.333 |
|  | **Strain** | **Experimental mouse’s features** | **Stimulus mice’s features** | **Environmental conditions** | **Housing conditions** | **Testing room** | **Isolation habituation** | **Control with a novel stimulus** | **Exposure time** | **Inter-trial interval** | **Number of individuals** | **Results (comparison of exploration time between familiar and unfamiliar conspecific in the second trial and ratio of exploration time over the total exposure duration in the first trial)** |
| (Ferguson et al., 2000) | EM: 129S7/SvEvBrd x C57BL/6J  SM: CD1 | -Age : 50 day-old | -Age : > 50 day-old (adults)  -Ovariectomized females | -12:12 LDC  -T : 23-24°C  -F&W ad lib | -EM & SM group-housed | ? | I = 7-10 days in HC | No | 5min | 30min | N=8 | P<0.05  R=0.5 – 0.133 |
| (Ferguson et al., 2001) | C57BL/6J x 129SvEv | -Age : 50-60 days | -Age > 50 day-old  -Ovariectomized females | -12:12 LDC  -L.on : 7am  -light phase  -T : 23°C  -F&W ad lib | -SM group-housed  -EM singly-housed | ? | I = 8-10 days  H= 12-18 hours in TR | yes | 5min | 30min | N=12 | P<0.05 |
| (Cohen et al., 2002) | FVB/N | -Age : 8-11 week-old | -Age : 23-29 day-old  -Males | -12:12 RLDC  -dark phase  -F&W ad lib | -EM group-housed (4-5) | -dim light | H=15min in TC | yes | 4min | 0, 5, 10, 15, 25min | N=48 | P<0.05 for ITI ≤ 15min  R=0.583 |
| (Wersinger et al., 2002) | EM:129/SvJ x C57BL/6  SM:129/Sv | -Age : 60-90 day-old  -socially naives | -Ovariectomized females | -12:12 LDC  -dark phase  -L.on : 6 a.m.  -F&W ad lib | -EM & SM singly housed | ? | ? | no | 5min | 30min | N=8 | P<0.05  R=0.3 – 0.167 |
| (Erbel-Sieler et al., 2004) | 129/SvEv x C57BL/6J | -Age : 17-20 week-old | -Age : Juveniles | -12:12 RDLC  -dark phase | -EM & SM group-housed | -Red or dim light | H= 1h in TR | no | 2min | 3 days | N=11 | P=0.03  R=0.5 – 0.25 |
| (Lim et al., 2007) | El & ddY | -Age : > 120 days | -Age : 25 – 35 days -Gender matched | -12 : 12 LDC  -L. on : 10pm  -dark phase  -T/H : 21°C/48%  -F&W ad lib except during encounters | EM group-housed | -Quiet -IR light | H = 30min | yes | 4min | 30min | N=8 | -El : failure -ddY : p<0.05 : R=0.417 – 0.292 |
|  | **Strain** | **Experimental mouse’s features** | **Stimulus mice’s features** | **Environmental conditions** | **Housing conditions** | **Testing room** | **Isolation habituation** | **Control with a novel stimulus** | **Exposure time** | **Inter-trial interval** | **Number of individuals** | **Results (comparison of exploration time between familiar and unfamiliar conspecific in the second trial and ratio of exploration time over the total exposure duration in the first trial)** |
| (Moretti, 2006) | EM: 129/SvEv  SM: C57BL/6J | -Naives | -Age : 3-4 week-old  -Males | ? | -TC: 498.75cm² | ? | H=15min in MC | no | 2min | 24h | N=15 | P<0.001  R=0.667 |
| (Lee et al., 2008) | EM:129/Sv  SM:BALB/c | -Age : 100-170 day-old | -Ovariectomized females | ? | -wait 2 weeks | ? | I>2 weeks | ? | ? | 30min | N=8-10 | P=0.007 |
| (Xikota et al., 2008) | 129/Sv x C57BL/6J | -Age : 12-14 week-old | -Age : juveniles  -Males | -12:12 LDC  -L.on : 7a.m  -F&W ad lib | -1428cm² | ? | EM : I >7days in MC  H>1h in the TR  SM : H=20min in MC | no | 5min | 30min | N>9 | R=0.333 – 0.25, p<0.05 |
| (Gao et al., 2009) | C57BL/6J | -Age : 8-12 week-old | -Age : 4-5 week-old  -Males | -12:12 LDC  -light phase  -F&W ad lib | -EM group-housed (4-5)  -MC ≠TC:522cm² | -dim light | H to the TR. H to the TC = 15min | no | 2min | 10, 30, 60, 120 min, or 1, 3, 7, 15, 30 days | N=10 | P<0.03 with ITI from 10min to 3 days  R=0.583 – 0.292 |
| (Rial et al., 2009b) | C57BL/6 | -Age : 12-14 week-old | -Age : juvenile  -Males | -12:12 LDC  -L.on : 7a.m  -F&W ad lib | -1428cm² | ? | EM : I >7 days in MC  SM : I=20min in MC | no | 5min | 30min | N=14 | R=0.4 – 0.3, p<0.5 |
| (Rial et al., 2009a) | 129/Sv x C57BL/6J | -Age : 3 and 11 month-old | -Age : 25-30 day-old  -Males | -12:12 LDC  -L.on : 7am  -F&W ad lib  -T : 22-24°C | -EM group-housed (4-5)  -1428cm² | ? | EM : I >7 days in MC  H>1h in the TR  SM : I=20min in MC | no | 5min | 30min | N>9 | -EM’s age=3months :  R=0.4 – 0.267 |
| (Sinai et al., 2010) | C57BL/6J | -Age : 8-14 week-old | -Age : 25-30 day-old  -Males | -12:12 LDC  -L.on : 5 a.m  -light phase  -F&W ad lib  -T/H : 20°C/50-60% | -EM & SM group-housed (5) | ? | H=15min in MC | yes | 2min | 20h | N=10 | R=0.597 – 0.39, p=0.007 |
| (Ito et al., 2011) | EM:C5BL/6J  SM:129S6 | -Age : 2-4 month-old | -Age : >6 week-old  -Ovariectomized females | -12:12 LDC  -F&W ad lib | ? | ? | I=2 weeks in HC | yes | 5min | 30min | ? | P<0.001 |
|  | **Strain** | **Experimental mouse’s features** | **Stimulus mice’s features** | **Environmental conditions** | **Housing conditions** | **Testing room** | **Isolation habituation** | **Control with a novel stimulus** | **Exposure time** | **Inter-trial interval** | **Number of individuals** | **Results (comparison of exploration time between familiar and unfamiliar conspecific in the second trial and ratio of exploration time over the total exposure duration in the first trial)** |
| (Prediger et al., 2011) | C57BL/6 | -Age : 3-4 month-old  -Naives | -Age : 25-30 day-old | -12:12 LDC  -L.on : 7 a.m  -light phase  -F&W ad lib  -T : 23°C | -EM group-housed (5)  -SM group-housed (10) | ? | SM : I=20min  EM : I=1h in HC | no | 5min | 30min | N=8 | P<0.0001, R=0.5 – 0.2 |
| (Samuelsen and Meredith, 2011) | C57BL/6 | -Age : 3 month-old  -Naives | ? | -reversed LDC  -dark phase  -F&W ad lib | -EM single-housed | -red light | I = full, in HC | yes – sep | 5min | 30min | N=24 | P<0.005 |
| (Kalkonde et al., 2011) | EM : C57BL/6J and DBA1/J SM : C57BL/6J | ? | -Age : 18-21 days -Males | -14 : 10 LDC  -F&W ad lib | -432cm² | -Quiet | H=30min MC | yes | 2min | 2h | N=8 | Significant differences (no pvalue) R=0.442 – 0.317 |
| (Suzuki et al., 2011) | C57BL/6N | ? | -Age : juvenile | -12:12 LDC  -light phase -F&W ad lib in HC | -EM group-housed (5-6)  -510cm² | -dim light | I=1h in MC | no | 45sec, 1min, 1.5min, 3min | 5min, 30min, 2h, 24h, 48h | N=8-30 | ET = 1.5min, ITI = 5min, 30min, 2h,: p<0.05  ET= 1min, ITI = 30min, 2h : p<0.05  ET=3min, ITI = 2h, p<0.05 |
| (Nomoto et al., 2012) | ? | ? | -Age : juvenile | -12:12 LDC  -light phase -F&W ad lib in HC | -EM group-housed (5-6)  -510cm² | -dim light | I= 1h in MC | no | 1.5min and 3min | 2h and 24h | N=11 | ET = 3min, ITI = 2h & 24h , ET =1.5min, ITI = 2h : P<0.05 |
| (Jacobs and Tsien, 2012) | C57BL/6 x CBA | -Age : 6-9 month-old | -Age : 1 month-old -Males | -12 : 12 LDC  -light phase  -F&W ad lib, except during encounters | -EM group-housed (3-5) | -quiet  -dim light | I = 24h & through the testing period in MC  OR I=0 : directly into the MC | yes - sep | 5min | 1h or 24h | N=15 | I=0, ITI = 1h : p=0.005  R=0.372 – 0.258  I=24h, ITI = 1h : p=0.03  R=0.427 – 0.328  I=0, ITI = 24h : p=0.02  R=0.328 – 0.243  I=24h, ITI = 24h : p=0.001  R=0.348 – 0.21  BALB/c, ITI = 1h : p=0.005  R=0.471 – 0.333 |
|  | **Strain** | **Experimental mouse’s features** | **Stimulus mice’s features** | **Environmental conditions** | **Housing conditions** | **Testing room** | **Isolation habituation** | **Control with a novel stimulus** | **Exposure time** | **Inter-trial interval** | **Number of individuals** | **Results (comparison of exploration time between familiar and unfamiliar conspecific in the second trial and ratio of exploration time over the total exposure duration in the first trial)** |
| (Yang et al., 2013) | C57BL/6 | -Age : 3-6 month-old | -Age : 3-6 month-old  -Males | -12:12 LDC -L.on : 7a.m | -EM group-housed  -536,4cm² | -dim light | H=15min in MC | yes | 5min | 30min, 3h, or 24h | N=8 | Significant differences for ITI = 30min : p<0.001 &3h : p<0.05 R=0.207 – 0.1 |
| (Hitti and Siegelbaum, 2014) | C57BL/6J | -Age : 10-12 week-old | -Age : 4-5 week-old  -Males | -12:12 LDC  -L.on : 6 am  -light phase  -F&W ad lib  -T : 21°C | -EM group-housed (2-5) | -dim light 12lux | H=1h in TR | yes - sep | 5min | 1h | N=15 | P<0.0001 – R=0.067 |
| (Bilkei-Gorzo et al., 2014) | SM : DBA/2J –Penk1 -/- | -Age : 3-5 months | -Age : 4 week-old  -Males | -14:10 LDC -L.on : 7p.m | -EM group-housed (3-5) |  | H=5min per day during 4 days, in TC | no | 5min | 1h 4h 8h 16h 24h | N=10-12 | P<0.001 for ITI = 1h or 4h |
| (Ishikawa et al., 2014) | C57BL/6N | -Age : 3 months | -Age : juvenile -Males | -12 : 12 LDC -light phase -F&W ad lib in HC | -EM group-housed (5-6) -510cm² |  | I= 1h in MC | no | 1.5min or 3min | 24h | N=10 à 22 | RDI = 0.6 : p<0.05 3min : R=0.444 – 0.278 |
| (Jacobs and Tsien, 2014) | C57BL/6J | -Age : 6-9 months | -Age : 1 mth  -Males  OR juvenile BALB/c | -12 : 12 LDC -F&W ad lib, except during encounters | EM group-housed | -quiet -dim light | H = 30min TC | yes | 5min | 1h or 24h | N=10 or 13 | ITI = 1h ; p=0.02 R=0.45 – 0.267  ITI = 24h ; p=0.003 R=0.333 – 0.20 |
| (da Rocha Lindner et al., 2015) | C57BL/6 | -Age : 3-4 month-old | -Age : juvenile | ? | -EM singly-housed  -HC: 1428cm²  -wait 3 days | ? | SM: I=20min  EM: H=24h in TR | no | 5min | 30min | N=6-7 | -Significant decline |

**Table III:** Summary of studies using the protocol social discrimination (b). We gathered information on the main parameters varying across laboratories and protocols. We also included a column for each protocol’s outcome in standard conditions, noting down the parameters for which the researchers obtained a significant p-value of investigation time decrease. Whenever possible, we also included in the results column, as a comparison and standardization element among protocols, the fraction of time spent by the experimental mouse exploring the first stimulus mouse in the first trial (R). Indeed, it is the only data that is always measured across all protocols. Abbreviations: LDC: Light-Dark Cycle; RLDC: Reversed Light-Dark Cycle; F: Food; W: Water; ad lib: ad libitum; EM: Experimental Mouse; SM: Stimulus Mouse; TC: Test Cage; H: Habituation; I=Isolation; TR: Test Room; MC: Mouse Cage; HC: Housing Cage; ITI: Inter-Trial Interval.

|  | **Strain** | **Experimental mouse’s features** | **Stimulus mice’s features** | **Environmental conditions** | **Housing conditions** | **Testing room** | **Isolation habituation** | **Exposure time** | **Inter-trial interval** | **Number of individuals** | **Results (comparison of exploration time between the familiar mouse and the unfamiliar mouse and ratio of exploration time over the total exposure duration in the first trial)** |
| --- | --- | --- | --- | --- | --- | --- | --- | --- | --- | --- | --- |
| (Marino et al., 2005) | EM : C57BL/6J x 129/SvEv SM : C57BL/6J | -Age : 3-6 month-old | -Ovariectomized female | -12:12 LDC  -L.on : 7 am  -light phase | -EM group-housed | ? | I > 1 week | 5min | 30min | N=11-13 | P=0.001  R=0.567 |
| (Richter et al., 2005) | C57BL/6JOlaHsd | -Age : 9-16 week-old | -Age : 25-30 day-old  -Gender : matched | -12:12 LDC  -L.on : 7 am  -light phase | -EM group-housed (5)  -HC:740cm²  -TC:280cm²  -wait 1 week | ? | I = 2h in TC | 4min | 60min or 24h | ? | P<0.001 for ITI = 60min, 24h  R=0.25 for ITI = 60min and R=0.104 for ITI = 24h |
| (Bielsky et al., 2005) | C57BL/6 | -Age : 2-5 month-old  -Sexually naives | -Age : Adult  -Ovariectomized Female | -14:10 LDC  -light phase  -F&W ad lib | -EM group-housed (3-5) | -T: 23-24°C | I=10daysin MC  H=1h in TR | 5min | 30min, 2h, 6h, 24h | N=10 | P<0.001 for ITI = 30min  R=0.367 |
| (Takayanagi et al., 2005) | EM:129/Sv x C57BL/6J SM:C57BL/6J | -Age : 4-7 month-old | -Ovariectomized female | ? | ? | ? | I=2days in MC | 5min | 30min | N=10 | P<0.05 |
| (Jia et al., 2006) | EM:ICR SM:C57BL/6 | -Age : 21 day-old | -Females | -12:12 LDC  -L.on : 7 am  -light phase  -F&W ad lib  -T/H : 23-25°C/40-60% | -EM group-housed (4-5) | ? | H=4min in TC | 4min | 24h | ? | P<0.01 |
|  | **Strain** | **Experimental mouse’s features** | **Stimulus mice’s features** | **Environmental conditions** | **Housing conditions** | **Testing room** | **Isolation habituation** | **Exposure time** | **Inter-trial interval** | **Number of individuals** | **Results (comparison of exploration time between the familiar mouse and the unfamiliar mouse and ratio of exploration time over the total exposure duration in the first trial)** |
| (Wanisch et al., 2008) | C57BL/6JOlaHsd | -Age : 13 week-old | -Age : 25-38 day-old  -Gender : matched | -12:12 LDC  -L.on : 7 am  -light phase | -EM group-housed (5)  -HC:740cm²  -TC:280cm²  -wait 1 week | ? | I = 2h in TC | 4min | 24h | ? | P<0.05  R=0.09 |
| (Jüch et al., 2009) | 129SV, C57BL/6 J, C57BL/6JOlaHsd, and  129SV x C57BL/6JOlaHsd | -Age : 25-36 week-old | -Age : 28-39 day-old  -Gender : matched | -12:12 LDC  -L.on : 7 am  -light phase  -F&W ad lib | -EM group-housed (5)  -HC:740cm²  -TC:280cm²  -wait 1 week | ? | I = 2h in TC | 4min | 1h, 3h, 6h, 24h | N=20 | P<0.05 for ITI = 1h, 3h, 6h, 24h |
| (Engelmann, 2009) | C57BL/6JOlaHsd | -Age : 9-16 week-old | -Age : 25-38 day-old  -Gender : matched | -12:12 LDC  -L.on : 7 am  -light phase | -EM group-housed (5)  -HC:740cm²  -TC:280cm²  -wait 1 week | -dim light 200lx | I = 2h in TC | 4min | 24h | N=15-21 | P<0.05 |
| (Garelick et al., 2009) | C57BL/6 | -Age : 3-4 month-old or 25-27 month-old | -Ovariectomized females | ? | ? | ? | ? | Sampling : (c) protocol (1min) | 10min, 3h, or 24h | ? | Significant difference 24h later for young mice, and only 3h later for old mice.  R=0.75 |
| (Noack et al., 2010) | C57BL/6JOlaHsd | -Age : 9-16 week-old | -Age : 25-35 day-old  -Gender : matched | -12:12 LDC  -L.on : 6 am  -light phase  -F&W ad lib | -EM group-housed (5)  -TC:352 cm² | ? | I = 2h in TC | 4min | 30min, 120min, 24h | N=10 | P<0.05 for ITI = 30min, 120min, 24h  R=0.16 |
| (Kasahara et al., 2011) | C57BL/6J | -Age : 18-35 week-old | -Age : 4-6 week-old  -Males | -12:12 LDC  -L.on : 6 am  -light phase  -T/H : 19-23°C/40-50%  -F&W ad lib | -EM group-housed (2-3)  -HC:264cm²  -TC:625cm² | ? | I= 3days  H=30min/day during 5 days in TC | Sampling : (c) protocol the day before  2min | 10min, 24h | ? | P<0.0001 for the difference novel-familiar for ITI=10min  R=0.333 |
|  | **Strain** | **Experimental mouse’s features** | **Stimulus mice’s features** | **Environmental conditions** | **Housing conditions** | **Testing room** | **Isolation habituation** | **Exposure time** | **Inter-trial interval** | **Number of individuals** | **Results (comparison of exploration time between the familiar mouse and the unfamiliar mouse and ratio of exploration time over the total exposure duration in the first trial)** |
| (Sánchez-Andrade and Kendrick, 2011) | C57BL/6 x 129Sv | -Age : 3-11 month-old | -Age : 3-11 month-old  -Gender : males  -sedated | -12:12 LDC  -L.on : 7 am  -light phase  -F&W ad lib | -EM group-housed (2-5)  -TC:1089cm² | ? | H=10min in TC | Sampling : (c) protocol the day before  2min | 24h | N=13-14 | p=0.001 |
| (McGowan et al., 2011) | EM:B6.129SF2/J  SM:C57BL/6J | -Age : 3-5 month-old  -Naives | -Ovariectomized female  -Age : 9 week-old | -dark phase | -EM & SM group-housed (3-5) | ? | In MC | 4min | 30min, 60min, 120min | N=12-16 | ITI=30min, p=0.009  ITI=60min, p=0.0001  R=0.458 |
| (Engelmann et al., 2011a) | 129S1SV, C57BL/6JOlaHsd and others | -Age : 8-16 week-old | -Age : 15-35 day-old  -Gender : matched | ? | -EM group-housed (3-5)  -SM group-housed  -352cm²  -H=2weeks | -quiet  -135-250 lux | -I=2h in MC | 4min | 24h | ? | P<0.01  R=0.125 |
| (Lukas et al., 2013) | C57BL/6N | ? | -Age : 3 week-old  -Males | -12:12 LDC  -L.on : 6 a.m.  -light phase  -T/H : 22°C/60%  -F&W ad lib | -EM & SM group-housed  -HC:1092cm²  -TC:690cm²  -wait > 1week | ? | ? | 4min | 60min | N=8-10 | P<0.005 |
| (Hädicke and Engelmann, 2013) | EM:129S1/SvImJ and C57BL/6JOlaHsd, and hybrids.  SM: C57BL/6JOlaHsd | -Age : 8-16 week-old  -Sexually naives | -Age : 25-35 day-old  -Gender : matched | -12:12 LDC  -L.on : 7 am  -light phase  -T/H : 22-24°C/55-65%  -F&W ad lib | -EM & SM group-housed (3-6)  -HC:740cm²  -TC:280cm²  -wait 1 week | ? | -I=2h in TC | 4min | 24h | N=19-24 | Significant differences for all strains  R=0.125 |
|  | **Strain** | **Experimental mouse’s features** | **Stimulus mice’s features** | **Environmental conditions** | **Housing conditions** | **Testing room** | **Isolation habituation** | **Exposure time** | **Inter-trial interval** | **Number of individuals** | **Results (comparison of exploration time between the familiar mouse and the unfamiliar mouse and ratio of exploration time over the total exposure duration in the first trial)** |
| (Wang et al., 2014) | C57BL/6 | - Age : 8 week-old | -8 week-old  -Males | -12:12 LDC  -L.on: 7:30 a.m.  -Light phase  -T/H : 20-24°C/40-70%  -F&W ad lib | ? | ? | ? | 4min | 30min | N=16 | Significant differences |
| (Kohl et al., 2015) | C57BL/6 | -Age : matched | ? | -12:12 LDC  -L.on: 7 a.m.  -Light phase  -ventilated cages  -T/H : 23°C/35-65%  -F&W ad lib | ? | ? | ? | ? | ? | N=14 | P<0.05  R=0.14 |
| (Bruining et al., 2015) | EM:C57BL/6J  SM:A/J | -Age : 3-5 month-old | -Age : matched  -Gender : matched | ? | ? | ? | H=5min in TC | 2min | 5min or 24h | N=10 | ITI=5min : p<0.01, ITI=24h : p<0.001 |
| (Perna et al., 2015) | C57BL/6JOlaHsd | -Age : 9-16 week-old | -Age : 25-38 day-old  -Gender : matched | -12:12 LDC  -L.on : 7 am  -light phase  -F&W ad lib | -EM group-housed (5)  -HC:740cm²  -wait 1 week | ? | -I=2h in MC | 4min | 24h | ? | Significant differences  R=0.167 |
| (James et al., 2015) | 129Sv x C57BL/6J | -Age : 3-5 month-old, and 18-24 month-old | -Adult  -Male  -Anesthetized | -12:12 LDC  -L.on : 7:30 am  -light phase  -F&W ad lib | -EM group-housed (2-5)  -TC:4050cm² | ? | H=10min in TC | Sampling: (c) protocol the day before.  2min | 24h | N=12-13 | P<0.05 for 3-5 month-old mice only |
| (Zhou et al., 2015) | C57BL/6 | -Age : 20 day-old | -Age : 20 day-old  -Females | -12:12 LDC  -T : 25°C  -F&W ad lib | -TC:400cm² | -quiet  -dim light | H=30min in TC | Sampling: (c) protocol 90min before.  5min | 90min | N=6 | Significant difference between novel and familiar on trial 5 |

**Table IV:** Summary of studies using the protocol habituation-dishabituation (c). We gathered information on the main parameters varying across laboratories and protocols. We also included a column for each protocol’s outcome in standard conditions, noting down the parameters for which the researchers obtained a significant p-value of investigation time decrease. Whenever possible, we also included in the result column, as a comparison and standardization element among protocols, the fraction of time spent by the experimental mouse exploring the first stimulus mouse in the first trial (R). Indeed, it is the only data that is always measured across all protocols. Abbreviations: LDC: Light-Dark Cycle; RLDC: Reversed Light-Dark Cycle; F: Food; W: Water; ad lib: ad libitum; EM: Experimental Mouse; SM: Stimulus Mouse; TC: Test Cage; H: Habituation; I=Isolation; TR: Test Room; MC: Mouse Cage; HC: Housing Cage; ITI: Inter-Trial Interval.

|  | **Strain** | **Experimental mouse’s features** | **Stimulus mice’s features** | **Environmental conditions** | **Housing conditions** | **Testing room** | **Isolation habituation** | **Control with a novel animal** | **Number of exposures during habituation** | **Exposure time** | **Inter-trial interval** | **Number of individuals** | **Results (report of comparison of exploration time between the repeated exposure sessions and ratio of exploration time over the total exposure duration in the first trial)** |
| --- | --- | --- | --- | --- | --- | --- | --- | --- | --- | --- | --- | --- | --- |
| (Dluzen and Kreutzberg, 1993) | CD1 | -Age : > 6 month-old | -Ovariectomized females | -12:12 LDC  -L.on : 6 am  -F&W ad lib | -EM singly-housed | ? | ? | Yes, 4^th^, 5^th^, and 6^th^ presentation | 3 | 2min | 20min | N=14 | Significant decline from T1 to T3. Significant decline from T4 to T6. Significant difference T3-T4. But significant difference T1-T4 (100 vs 60).  R=0.833 |
| (Winslow and Camacho, 1995) | CD1 | ? | -Age : > 40 day-old  -Ovariectomized females  OR -Males  OR  -Cage-mate ovariectomized females | -12-12 LDC  -L.on : 6 am  -light phase  -T : 24°C  -F&W ad lib | -SM: group-housed (5) | ? | I= 1-2 weeks in HC | Yes, 5^th^ presentation (either the same or a novel) | 4 | 1min | 10min | N=8-10 | SM= Ovariectomized females : Significant decline T1-T4, and significant difference T4-T5, but also significant difference T1-T5.  0.75  SM= Males: Significant difference T1-T2, but constant investigation time T2-T4. No 5^th^ trial.  R=0.333  SM=Cage-mate ovariectomized females : significant decline T1-T4, No 5^th^ trial  R=0.333 |
| (Ferguson et al., 2000) | EM: 129S7/SvEvBrd x C57BL/6J  SM: CD1 | -Age : 50 day-old | -Age : > 50 day-old (adults)  -Ovariectomized females | -12:12 LDC  -T : 23-24°C  -F&W ad lib | -EM & SM group-housed | ? | I = 7-10 days in HC | Yes, 5^th^ presentation | 4 | 1min | 10min | N=18 | Significant decrease T1-T4, significant difference T4-T5, but significant difference T1-T5.  R=0.667 |
| (Jin et al., 2007) | ? | -Age : 10 week-old  -Sexually naives | -Females | ? | ? | ? | I = 7-10 days in HC | Yes, 5^th^ presentation | 4 | 1min | 10min | N=10 | Significant decline T1-T4. No significant differences T1-T5  R=0.833 |
|  | **Strain** | **Experimental mouse’s features** | **Stimulus mice’s features** | **Environmental conditions** | **Housing conditions** | **Testing room** | **Isolation habituation** | **Control with a novel animal** | **Number of exposures during habituation** | **Exposure time** | **Inter-trial interval** | **Number of individuals** | **Results (report of comparison of exploration time between the repeated exposure sessions and ratio of exploration time over the total exposure duration in the first trial)** |
| (Wersinger et al., 2002) | EM: 129/SvJ x C57BL/6J  SM: 129/Sv | -Socially naives | -Ovariectomized females  -Socially naives | -dark phase  -F&W ad lib | ? | ? | ? | Yes, 11^th^ presentation | 10 | 1min | 5min | N=8 | No significant difference between T1 and T2, but significant decline T1-T10 (from T4). No significant difference between T1 and T11.  R=0.417 |
| (Imwalle et al., 2002) | EM: C57BL/6J  SM: CD1 | -Age : 50 day-old  -Sexually naives | -Age : adults, > 45 day-old  -Ovariectomized females | -12:12 LDC  -L.on : 7 am  -light phase  -T : 22-24°C  -F&W ad lib | -EM group-housed | ? | I= from the age of 40 day-old (10 days), in HC | Yes, 9^th^ presentation | 8 | 1min | 10min | N=10 | Increase in the investigation duration from T1 to T4. Then, significant decrease, so, significant difference T1-T8. No significant difference between T1 and T9.  R=0.667 |
| (Bielsky et al., 2004) | C57BL/6 x 129/SvJ | -Age : 2-5 month-old  -Sexually naives | -Ovariectomized females | -14:10 LDC  -light phase  -F&W ad lib | -EM group-housed (3-5) | -23-24°C  -300-400lx | I=10days  H=1h in TR | Yes, 5th presentation | 4 | 1min | 10min | N=8 | P<0.01 for the difference T1-T3 , T1-T4, and T4-T5  R=0.833 |
| (Long et al., 2004) | EM: 129S6  SM: CD1 | -Age : 3-8 month-old | -Ovariectomized females | -12:12 LDC  -T : 22°C  -light phase | ? | ? | I= 2 weeks | Yes, 5^th^ presentation | 4 | 1min | 10min | N=9 | No significant difference T1-T2, but significant decline T2-T4. No significant difference T1-T5. R=0.417 |
| (Marino et al., 2005) | EM : C57BL/6J x 129/SvEv SM : C57BL/6J | -Age : 3-6 month-old | -Ovariectomized female | -12:12 LDC  -L.on : 7 am  -light phase | -EM group-housed | ? | I > 1 week | Yes, 5^th^ presentation | 4 | 1min | 10min | N=12-13 | P<0.01 for the decrease T1-T4. No significant differences T1-T5.  R=0.833 |
| (Bielsky et al., 2005) | C57BL/6 x 129/SvJ | -Age : 2-5 month-old  -Sexually naives | -Age : adult  -Ovariectomized female | -14:10 LDC  -light phase  -F&W ad lib | -EM group-housed (3-5) | -23-24°C | I= 10 days  H=1h in TR | Yes, 5^th^ presentation | 4 | 1min | 10min | N=8 | Significant decrease during habituation (p<0.001 T1-T4), and no significant difference between T1 and T5.  R=0.717 |
|  | **Strain** | **Experimental mouse’s features** | **Stimulus mice’s features** | **Environmental conditions** | **Housing conditions** | **Testing room** | **Isolation habituation** | **Control with a novel animal** | **Number of exposures during habituation** | **Exposure time** | **Inter-trial interval** | **Number of individuals** | **Results (report of comparison of exploration time between the repeated exposure sessions and ratio of exploration time over the total exposure duration in the first trial)** |
| (Wersinger et al., 2007) | EM: 129/SvJ x C57BL/6J  SM: 129S1/  SvImJ/Cr | -Age : 60-90 day-old | -Ovariectomized female | -12:12 LDC  -light phase  -F&W ad lib | -EM & SM group-housed | ? | I=20 days | Yes, 5^th^ presentation | 4 | 1min | 10min | N=9 | Significant decrease during habituation, and significant differences between T4 and T5.  R=0.667 |
| (Vulih-Shultzman et al., 2007) | 129/Sv x C57BL/6 | -Age : 3 month-old | -Age : 18 month-old  -Female | -12:12 LDC  -F&W ad lib |  |  |  | Yes, 6^th^ presentation | 5 | 5min | 30min | N=4 | P<0.01 for the decrease T1-T2. Significant decrease T1-T5. P<0.01 for the increase T5-T6, but also significant difference T1-T6. |
| (Scearce-Levie et al., 2008) | EM: 129X1/SvJ x C57BL/6NCrl  SM: C57BL/6J | -Age : 4-8 month-old | -Ovariectomized females | -12:12 LDC  -light phase  -F&W ad lib | -EM & SM group-housed | 350 lux | I > 5 days in HC  H=3min in TR | Yes, 11^th^ presentation | 10 | 1.5min | 3min | N=14 | Significant decrease during habituation, and significant differences between T10 and T11 (p<0.05)  R=0.667 |
| (Lee et al., 2008) | EM:129/Sv  SM:BALB/c | -Age : 100-170 day-old | -Ovariectomized females | ? | -wait 2 weeks |  | I>2 weeks | Yes, 5^th^ presentation | 4 | ? | 10min | N=8-10 | No significant decrease during habituation, but significant difference between T4 and T5  P=0.007 |
| (Savonenko et al., 2008) | EM:C57BL/6J  SM: Swiss-Webster or C57BL/6J | -Age : 4-8 month-old | -Age : 23-28 day-old  -Males | -light phase | ? | 23-24 °C | H=1h in TR  H=2min in MC | Yes, 4^th^ presentation | 3 | 2min | 20min | ? | Significant decrease during habituation, and difference between T3-T4 for the C57BL/6 juvenile mice only.  R=0.6 |
| (Bégou et al., 2008a) | BALB/c/129Sv | -Age: 3-5 month-old | -Anesthetized mice | -12:12 LDC  -L.on : 6am  -light phase  -T : 21-23°C  -F&W ad lib | -EM group-housed (8)  -SM group-housed (6)  -HC=TC:200cm² | ? | I=1 week  H=1h in TR | Yes, 4^th^ presentation | 3 | 3min | 12min | N=8 | P<0.001 for the decrease during habituation, p<0.05 for the difference T3-T4.  R=0.222 |
|  | **Strain** | **Experimental mouse’s features** | **Stimulus mice’s features** | **Environmental conditions** | **Housing conditions** | **Testing room** | **Isolation habituation** | **Control with a novel animal** | **Number of exposures during habituation** | **Exposure time** | **Inter-trial interval** | **Number of individuals** | **Results (report of comparison of exploration time between the repeated exposure sessions and ratio of exploration time over the total exposure duration in the first trial)** |
| (Pierman et al., 2008) | C57BL/6J | ? | -Ovariectomized female | -12:12 RLDC  -dark phase  -F&W ad lib  -T : 22°C | -EM singly-housed | ? | -I > 1 week | 5^th^presentation:half = novel, half=familiar | 4 | 1min | 10min | N=10 | P<0.0001 for the difference T1-T4  P<0.05 for the difference T4-T5 Novel  R=0.917 |
| (Satomoto et al., 2009) | C57BL/6 | -Age : 18-week-old | -females | -12:12 LDC  -L.on : 7am  -T : 20-22°C  -F&W ad lib | -EM group-housed (4) | ? | -I=7days | Yes, 5^th^ presentation | 4 | 1min | 10min | N=8 | Significant differences between T1 and T3 or T4. T1 similar to T5.  R=0.867 |
| (Zou et al., 2009) | CD1 | -Age : 10 week-old | -Age : 4 week-old | -12:12 LDC  -F&W ad lib | -EM singly-housed  -SM group-housed (2-3)  -TC:360cm² | fluorescent light 5W | -H= 5 days in TR  -H=15min in TC | Yes, 5^th^ presentation | 4 | 2min | 10min | N=14 | P<0.05 for the difference between T1 and T4. T1 similar to T5 : p>0.05  R=0.917 |
| (Garelick et al., 2009) | C57BL/6 | -Age : 3-4 month-old or 25-27 month-old | -Ovariectomized females | ? | ? | ? | ? | Yes, 7^th^ presentation | 6 | 1min | 10min, and 3h or 24h after: duplication of T5 with a fresh novel mouse | ? | Significant decrease during habituation, and for the difference T6-T7 for both young and old mice. Significant difference novel-familiar 24h later for young mice, and 3h later for old mice.  R=0.75 |
| (Glynn et al., 2010) | 129Ola/C57Bl6 | -Age : 4 month-old | -Females, or juvenile males | -12:12 LDC  -light phase  -T/H : 21-23°C/45-65%  -F&W ad lib | -EM group-housed (9-16) |  | H=10min in TC | Yes, 5^th^ presentation | 4 | 1min | 10min | N=8 | P<0.001 for the decrease during habituation, significant difference between T4 and T5  R=0.75 |
| (Lan et al., 2011) | SM: C57BL/6 | -Age : 10 week-old | -Ovariectomized females | ? | -SM singly-housed | ? | I = 1 week | Yes, 5^th^ presentation; and 6^th^ with the familiar mouse again | 4 | 1min | 10min | N=7 | Significant decrease T1-T4, significant difference T4-T5, and T5-T6 |
|  | **Strain** | **Experimental mouse’s features** | **Stimulus mice’s features** | **Environmental conditions** | **Housing conditions** | **Testing room** | **Isolation habituation** | **Control with a novel animal** | **Number of exposures during habituation** | **Exposure time** | **Inter-trial interval** | **Number of individuals** | **Results (report of comparison of exploration time between the repeated exposure sessions and ratio of exploration time over the total exposure duration in the first trial)** |
| (Sánchez-Andrade and Kendrick, 2011) | C57BL/6 x 129Sv | -Age : 3-11 month-old | -Age : 3-11 month-old  -Gender : males  -sedated | -12:12 LDC  -L.on : 7 am  -light phase  -F&W ad lib | -EM group-housed (2-5)  -TC:1089cm² | ? | H=10min in TC | Yes, 5^th^ presentation | 4 | 1min | 10min, and 24h after: duplication of T5 with a fresh novel mouse | N=13-14 | Significant decrease during habituation, and for the difference between T4 and T5. Significant difference between novel and familiar 24h later. |
| (Kasahara et al., 2007) | C57BL/6J | -Age : 18-35 week-old | -Age : 4-6 week-old  -Males | -12:12 LDC  -L.on : 6 am  -light phase  -T/H : 19-23°C/40-50%  -F&W ad lib | -EM group-housed (2-3)  -HC:264cm²  -TC:625cm² | ? | I= 3days  H=30min/day during 5 days in TC | Yes, 5^th^ presentation | 4 | 2min | 10min, and between T4-T5, 10min or 24h | ? | P<0.0001 for the difference novel-familiar during T5 for ITI=10min  R=0.333 |
| (Sakamoto et al., 2011) | C57BL/6J | -Age : 4-month-old and 12-month-old | -Males | -12:12 LDC  -L.on : 6am | -TC : 300cm² | ? | H=30 in TC | Yes, 5th presentation | 4 | 1min | 10min | ? | Significant decrease during habituation for both 4 and 12 month-old mice, p<0.05 and p<0.01 for the difference T4-T5 for 4 and 12 month-old mice, respectively.  R=0.333 |
| (Shiryaev et al., 2011) | C57BL/6 x BALB/c | -Age : 5 month-old | -Age : 18 month-old  -Female | -14:10 LDC  -L.on : 5am  -light phase  -F&W ad lib | ? | ? | ? | Yes, 6^th^ presentation | 5 | 5min | 30min | N=4-6 | Decrease T1-T5 but significant only from between T1-T4. No significant difference between T1 and T6. |
| (Koliatsos et al., 2011) | C57BL/6J | ? | -Age : 23-28 day-old  -Males | -light phase | Wait 1 week | 23-24°C | H= 1h in TR  H= 2min in MC | Yes, 4^th^ presentation | 3 | 2min | 20min | N=7 | Significant decrease (p<0.0001) T1-T3, significant difference (p<0.001) T3-T4. No significant differences T1-T4  R=0.542 |
|  | **Strain** | **Experimental mouse’s features** | **Stimulus mice’s features** | **Environmental conditions** | **Housing conditions** | **Testing room** | **Isolation habituation** | **Control with a novel animal** | **Number of exposures during habituation** | **Exposure time** | **Inter-trial interval** | **Number of individuals** | **Results (report of comparison of exploration time between the repeated exposure sessions and ratio of exploration time over the total exposure duration in the first trial)** |
| (Kercmar et al., 2011) | C57BL/6J | -Age : 80 day-old | -Ovariectomized females | -12:12 LDC  -L.on : 5am  -dark phase  -F&W ad lib | -EM group-housed (3), or singly-housed at PN30 or singly-housed at PN30 and group-housed at PN60.  -HC:836cm² or 525cm² | -red light | I = 24h in MC for the group-housed mice | Yes, 9^th^ presentation | 8 | 1min | 9min | ? | P<0.001 for both the difference T1-T8 and the difference T8-T9, for the group-housed males.  P<0.01 for the difference T1-T8 and the difference T8-T9, for the isolated/group-housed males.  R=0.667 |
| (Faizi et al., 2011) | EM: 2N  SM: C57BL/6J | -Age : 9 or 18 month-old | -4-6 month-old males, or ovariectomized females | -12:12 LDC  -light phase  -F&W ad lib | -SM singly-housed | ? | ? | Yes, 5^th^ presentation | 4 | 1min | 10min | N=9 | P<0.0001 for the difference between T1 and T4, and between T4 and T5 |
| (Faizi et al., 2012) | C57BL/6J | -Age : 5-6 month-old | -Age : 4 month-old  -Ovariectomized females | -12:12 LDC  -light phase  -F&W ad lib | -EM singly-housed | ? | H=2h in TR | Yes, 5^th^ presentation ; and 6^th^ with the familiar mouse again | 4 | 1min | 10min | N=14 | Significant decrease during habituation (p=0.0014), significant difference T4-T5 (p<0.0001), and T5-T6 (p<0.0001)  R=0.7 |
| (Jacobs and Tsien, 2012) | C57BL/6 x CBA | -Age : 6-9 month-old | -Age : 1 month-old -Males | -12 : 12 LDC  -light phase  -F&W ad lib, except during encounters | -EM group-housed (3-5) | -quiet  -dim light | H=15min in MC | Yes, 5^th^ presentation | 4 | 1min | 10min | N=10-12 | Significant differences between T1-T3. T5 didn’t reach the same level of investigation as T1. |
| (Klemenhagen et al., 2013) | C57BL/6J | -Age : 7-10 week-old | -Age : 7-10 week-old  -Males | -12:12 LDC  -L.on : 6 am  -dark phase  -T : 20-22°C  -F&W ad lib | -EM group-housed (2-5)  -wait 1 week | ? | H>1h in TR | Yes, 10^th^ presentation | 9 | 1min | 5min | N=14 | Significant differences between T1-T9 and T9-T10  R=0.5 |
| (Nelson et al., 2013) | EM:C57BL/6 SM:129X1/SvJ | ? | -Ovariectomized females | ? | -EM group-housed | -dim light | I=7 days | Yes, 5^th^ presentation | 4 | 1min | 10min | N=15 | No decrease during habituation, but significant difference between T4 and T5 |
|  | **Strain** | **Experimental mouse’s features** | **Stimulus mice’s features** | **Environmental conditions** | **Housing conditions** | **Testing room** | **Isolation habituation** | **Control with a novel animal** | **Number of exposures during habituation** | **Exposure time** | **Inter-trial interval** | **Number of individuals** | **Results (report of comparison of exploration time between the repeated exposure sessions and ratio of exploration time over the total exposure duration in the first trial)** |
| (Greco et al., 2013) | C57BL/6J | -Age : 2 month-old or 6 month-old | ? | -12:12 LDC  -L.on : 7am  -light phase  -F&W ad lib  -T : 21°C | -EM group-housed (2-4)  -TC:1130.5cm² | -150-180 lux | H=1h in TR  H=30min in TC | Yes, 5^th^ presentation | 4 | 2min | 10min | N=11-12 | P<0.005 and p<0.01 for the difference between T1 and T4 and for 2 and 6 month-old mice  P=0.01 for the difference T4-T5 for all experimental mice |
| (Jacobs and Tsien, 2014) | C57BL/6J | -Age : 6-9 months-old | -Age : 1 month-old  -Males | -12 : 12 LDC -F&W ad lib, except during encounters | -EM group-housed | -quiet -dim light | H = 30min TC | Yes, 5th presentation | 4 | 1min | 10min | N=10 or 13 | T1-T2 : p=0.001  T2-T3 : p=0.0003  R=0.7 |
| (Hitti and Siegelbaum, 2014) | EM : C57BL/6J  SM : CD1 | -Age : 10-12 week-old | -Age : 10 week-old  -Ovariectomized females | -12:12 LDC  -L.on : 6 am  -light phase  -F&W ad lib  -T : 21°C | -EM group-housed (2-5) | -dim light 12lux | I= 7 days in HC | Yes, 5^th^ presentation | 4 | 1min | 10min | N=15 | Significant difference between T1 and T2, but no further decrease from T2 to T4. Then, significant difference between T4 and T5.  R=0.3 |
| (James et al., 2015) | 129Sv x C57BL/6J | -Age : 3-5 month-old, and 18-24 month-old | -Adult  -Male  -Anesthetized | -12:12 LDC  -L.on : 7:30 am  -light phase  -F&W ad lib | -EM group-housed (2-5)  -TC:4050cm² | ? | H=10min in TC | Yes, 5^th^ presentation | 4 | 1min | 10min | N=12-13 | P<0.001 for the habituation for both the 3-5 and the 18-24 month-old mice  P<0.05 for the difference novel-familiar (T5) only for the 3-5 month-old mice |
| (Zhou et al., 2015) | C57BL/6 | -Age : 20 day-old | -Age : 20 day-old  -Females | -12:12 LDC  -T : 25°C  -F&W ad lib | -TC:400cm² | -quiet  -dim light | H=30min in TC | Yes, 5^th^ presentation | 4 | 5min | 30min but 90 min between T4 and T5 | N=6 | Significant difference between T1-T4, and between novel and familiar on trial 5 |

**References**

Bégou, M., Volle, J., Bertrand, J.-B., Brun, P., Job, D., Schweitzer, A., et al. (2008a). The stop null mice model for schizophrenia displays cognitive and social deficits partly alleviated by neuroleptics. *Neuroscience* 157, 29–39. doi:10.1016/j.neuroscience.2008.07.080.

Bégou, M., Volle, J., Bertrand, J.-B., Brun, P., Job, D., Schweitzer, A., et al. (2008b). The stop null mice model for schizophrenia displays cognitive and social deficits partly alleviated by neuroleptics. *Neuroscience* 157, 29–39. doi:10.1016/j.neuroscience.2008.07.080.

Bielsky, I. F., Hu, S.-B., Ren, X., Terwilliger, E. F., and Young, L. J. (2005). The V1a vasopressin receptor is necessary and sufficient for normal social recognition: a gene replacement study. *Neuron* 47, 503–13. doi:10.1016/j.neuron.2005.06.031.

Bielsky, I. F., Hu, S.-B., Szegda, K. L., Westphal, H., and Young, L. J. (2004). Profound Impairment in Social Recognition and Reduction in Anxiety-Like Behavior in Vasopressin V1a Receptor Knockout Mice. *Neuropsychopharmacology* 29, 483–493. doi:10.1038/sj.npp.1300360.

Bilkei-Gorzo, A., Mauer, D., Michel, K., and Zimmer, A. (2014). Dynorphins regulate the strength of social memory. *Neuropharmacology* 77, 406–413. doi:10.1016/j.neuropharm.2013.10.023.

Bluthé, R. M., Gheusi, G., and Dantzer, R. (1993). Gonadal steroids influence the involvement of arginine vasopressin in social recognition in mice. *Psychoneuroendocrinology* 18, 323–35. doi:10.1016/0306-4530(93)90028-J.

Brennan, P. A., and Kendrick, K. M. (2006). Mammalian social odours: attraction and individual recognition. *Philos. Trans. R. Soc. B-Biological Sci.* 361, 2061–2078. doi:10.1098/rstb.2006.1931.

Bruining, H., Matsui, A., Oguro-Ando, A., Kahn, R. S., van‘t Spijker, H. M., Akkermans, G., et al. (2015). Genetic Mapping in Mice Reveals the Involvement of Pcdh9 in Long-Term Social and Object Recognition and Sensorimotor Development. *Biol. Psychiatry* 78, 485–495. doi:10.1016/j.biopsych.2015.01.017.

Choleris, E., Clipperton-Allen, A. E., Phan, A., and Kavaliers, M. (2009). Neuroendocrinology of social information processing in rats and mice. *Front. Neuroendocrinol.* 30, 442–459. doi:10.1016/j.yfrne.2009.05.003.

Choleris, E., Kavaliers, M., and Pfaff, D. W. (2004). Functional Genomics of Social Recognition. *J. Neuroendocrinol.* 16, 383–389. doi:10.1111/j.0953-8194.2004.01178.x.

Cohen, O., Erb, C., Ginzberg, D., Pollak, Y., Seidman, S., Shoham, S., et al. (2002). Neuronal overexpression of “readthrough” acetylcholinesterase is associated with antisense-suppressible behavioral impairments. *Mol. Psychiatry* 7, 874–885. doi:10.1038/sj.mp.4001103.

da Rocha Lindner, G., Bonfanti Santos, D., Colle, D., Gasnhar Moreira, E. L., Daniel Prediger, R., Farina, M., et al. (2015). Improved neuroprotective effects of resveratrol-loaded polysorbate 80-coated poly(lactide) nanoparticles in MPTP-induced Parkinsonism. *Nanomedicine* 10, 1127–1138. doi:10.2217/nnm.14.165.

de Chaumont, F., Ey, E., Torquet, N., Lagache, T., Dallongeville, S., Imbert, A., et al. (2018). Live Mouse Tracker: real-time behavioral analysis of groups of mice. *bioRxiv*. doi:https://doi.org/10.1101/345132.

Dluzen, D. E., and Kreutzberg, J. D. (1993). 1-Methyl-4-phenyl-1,2,3,6-tetrahydropyridine (MPTP) disrupts social memory/recognition processes in the male mouse. *Brain Res.* 609, 98–102. Available at: http://www.ncbi.nlm.nih.gov/pubmed/8099527 [Accessed June 6, 2018].

Engelmann, M. (2009). Competition between two memory traces for long-term recognition memory. *Neurobiol. Learn. Mem.* 91, 58–65. doi:10.1016/J.NLM.2008.08.009.

Engelmann, M., Hädicke, J., and Noack, J. (2011a). Testing declarative memory in laboratory rats and mice using the nonconditioned social discrimination procedure. *Nat. Protoc.* 6, 1152–1162. doi:10.1038/nprot.2011.353.

Engelmann, M., Hädicke, J., and Noack, J. (2011b). Testing declarative memory in laboratory rats and mice using the nonconditioned social discrimination procedure. *Nat. Protoc.* 6, 1152–1162. doi:10.1038/nprot.2011.353.

Engelmann, M., Wotjak, C. T., and Landgraf, R. (1995). Social discrimination procedure: an alternative method to investigate juvenile recognition abilities in rats. *Physiol. Behav.* 58, 315–21. Available at: http://www.ncbi.nlm.nih.gov/pubmed/7568435 [Accessed June 6, 2018].

Erbel-Sieler, C., Dudley, C., Zhou, Y., Wu, X., Estill, S. J., Han, T., et al. (2004). Behavioral and regulatory abnormalities in mice deficient in the NPAS1 and NPAS3 transcription factors. *Proc. Natl. Acad. Sci. U. S. A.* 101, 13648–53. doi:10.1073/pnas.0405310101.

Faizi, M., Bader, P. L., Saw, N., Nguyen, T.-V. V., Beraki, S., Wyss-Coray, T., et al. (2012). Thy1-hAPP ^Lond/Swe+^ mouse model of Alzheimer’s disease displays broad behavioral deficits in sensorimotor, cognitive and social function. *Brain Behav.* 2, 142–154. doi:10.1002/brb3.41.

Faizi, M., Bader, P. L., Tun, C., Encarnacion, A., Kleschevnikov, A., Belichenko, P., et al. (2011). Comprehensive behavioral phenotyping of Ts65Dn mouse model of Down Syndrome: Activation of β1-adrenergic receptor by xamoterol as a potential cognitive enhancer. *Neurobiol. Dis.* 43, 397–413. doi:10.1016/j.nbd.2011.04.011.

Ferguson, J. N., Aldag, J. M., Insel, T. R., and Young, L. J. (2001). Oxytocin in the medial amygdala is essential for social recognition in the mouse. *J. Neurosci.* 21, 8278–85. Available at: http://www.ncbi.nlm.nih.gov/pubmed/11588199 [Accessed June 5, 2018].

Ferguson, J. N., Young, L. J., Hearn, E. F., Matzuk, M. M., Insel, T. R., and Winslow, J. T. (2000). Social amnesia in mice lacking the oxytocin gene. *Nat. Genet.* 25, 284–288. doi:10.1038/77040.

Ferguson, J. N., Young, L. J., and Insel, T. R. (2002). The Neuroendocrine Basis of Social Recognition. *Front. Neuroendocrinol.* 23, 200–224. doi:10.1006/frne.2002.0229.

Gao, X.-M., Elmer, G. I., Adams-Huet, B., and Tamminga, C. A. (2009). Social memory in mice: disruption with an NMDA antagonist and attenuation with antipsychotic drugs. *Pharmacol. Biochem. Behav.* 92, 236–42. doi:10.1016/j.pbb.2008.11.016.

Garelick, M. G., Chan, G. C. K., DiRocco, D. P., and Storm, D. R. (2009). Overexpression of Type I Adenylyl Cyclase in the Forebrain Impairs Spatial Memory in Aged But Not Young Mice. *J. Neurosci.* 29, 10835–10842. doi:10.1523/JNEUROSCI.0553-09.2009.

Glynn, D., Gibson, H. E., Harte, M. K., Reim, K., Jones, S., Reynolds, G. P., et al. (2010). Clorgyline-mediated reversal of neurological deficits in a Complexin 2 knockout mouse. *Hum. Mol. Genet.* 19, 3402–3412. doi:10.1093/hmg/ddq252.

Greco, B., Managò, F., Tucci, V., Kao, H.-T., Valtorta, F., and Benfenati, F. (2013). Autism-related behavioral abnormalities in synapsin knockout mice. *Behav. Brain Res.* 251, 65–74. doi:10.1016/j.bbr.2012.12.015.

Hädicke, J., and Engelmann, M. (2013). Social Investigation and Long-Term Recognition Memory Performance in 129S1/SvImJ and C57BL/6JOlaHsd Mice and Their Hybrids. *PLoS One* 8, e54427. doi:10.1371/journal.pone.0054427.

Hitti, F. L., and Siegelbaum, S. A. (2014). The hippocampal CA2 region is essential for social memory. *Nature* 508, 88–92. doi:10.1038/nature13028.

Imwalle, D. B., Scordalakes, E. M., and Rissman, E. F. (2002). Estrogen receptor alpha influences socially motivated behaviors. *Horm. Behav.* 42, 484–91. Available at: http://www.ncbi.nlm.nih.gov/pubmed/12488114 [Accessed June 6, 2018].

Ishikawa, R., Kim, R., Namba, T., Kohsaka, S., Uchino, S., and Kida, S. (2014). Time-dependent enhancement of hippocampus-dependent memory after treatment with memantine: Implications for enhanced hippocampal adult neurogenesis. *Hippocampus* 24, 784–793. doi:10.1002/hipo.22270.

Ito, W., Chehab, M., Thakur, S., Li, J., and Morozov, A. (2011). BDNF-restricted knockout mice as an animal model for aggression. *Genes, Brain Behav.* 10, 365–374. doi:10.1111/j.1601-183X.2010.00676.x.

Jacobs, S. A., and Tsien, J. Z. (2012). Genetic Overexpression of NR2B Subunit Enhances Social Recognition Memory for Different Strains and Species. *PLoS One* 7, e36387. doi:10.1371/journal.pone.0036387.

Jacobs, S. A., and Tsien, J. Z. (2014). Overexpression of the NR2A subunit in the forebrain impairs long-term social recognition and non-social olfactory memory. *Genes. Brain. Behav.* 13, 376–84. Available at: http://www.ncbi.nlm.nih.gov/pubmed/24834524 [Accessed June 6, 2018].

James, B. M., Li, Q., Luo, L., and Kendrick, K. M. (2015). Aged neuronal nitric oxide knockout mice show preserved olfactory learning in both social recognition and odor-conditioning tasks. *Front. Cell. Neurosci.* 9, 105. doi:10.3389/fncel.2015.00105.

Jia, F., Kato, M., Dai, H., Xu, A., Okuda, T., Sakurai, E., et al. (2006). Effects of histamine H3 antagonists and donepezil on learning and mnemonic deficits induced by pentylenetetrazol kindling in weanling mice. *Neuropharmacology* 50, 404–411. doi:10.1016/j.neuropharm.2005.09.017.

Jin, D., Liu, H.-X., Hirai, H., Torashima, T., Nagai, T., Lopatina, O., et al. (2007). CD38 is critical for social behaviour by regulating oxytocin secretion. *Nature* 446, 41–45. doi:10.1038/nature05526.

Johnson, Z. V, and Young, L. J. (2015). Neurobiological mechanisms of social attachment and pair bonding. *Curr. Opin. Behav. Sci.* 3, 38–44. doi:10.1016/j.cobeha.2015.01.009.

Jüch, M., Smalla, K.-H., Kähne, T., Lubec, G., Tischmeyer, W., Gundelfinger, E. D., et al. (2009). Congenital lack of nNOS impairs long-term social recognition memory and alters the olfactory bulb proteome. *Neurobiol. Learn. Mem.* 92, 469–484. doi:10.1016/j.nlm.2009.06.004.

Kalkonde, Y. V., Shelton, R., Villarreal, M., Sigala, J., Mishra, P. K., Ahuja, S. S., et al. (2011). The CC chemokine receptor 5 regulates olfactory and social recognition in mice. *Neuroscience* 197, 153–161. doi:10.1016/j.neuroscience.2011.09.039.

Kasahara, M., Groenink, L., Breuer, M., Olivier, B., and Sarnyai, Z. (2007). Altered behavioural adaptation in mice with neural corticotrophin-releasing factor overexpression. *Genes, Brain Behav.* 6, 598–607. doi:10.1111/j.1601-183X.2006.00286.x.

Kasahara, M., Groenink, L., Kas, M. J. H., Bijlsma, E. Y., Olivier, B., and Sarnyai, Z. (2011). Influence of transgenic corticotropin-releasing factor (CRF) over-expression on social recognition memory in mice. *Behav. Brain Res.* 218, 357–362. doi:https://doi.org/10.1016/j.bbr.2010.12.029.

Kavaliers, M., Choleris, E., Ågmo, A., and Pfaff, D. W. (2004). Olfactory-mediated parasite recognition and avoidance: linking genes to behavior. *Horm. Behav.* 46, 272–283. doi:10.1016/j.yhbeh.2004.03.005.

Kercmar, J., Büdefeld, T., Grgurevic, N., Tobet, S. A., and Majdic, G. (2011). Adolescent social isolation changes social recognition in adult mice. *Behav. Brain Res.* 216, 647–651. doi:10.1016/j.bbr.2010.09.007.

Klemenhagen, K. C., O’Brien, S. P., and Brody, D. L. (2013). Repetitive Concussive Traumatic Brain Injury Interacts with Post-Injury Foot Shock Stress to Worsen Social and Depression-Like Behavior in Mice. *PLoS One* 8, e74510. doi:10.1371/journal.pone.0074510.

Kogan, J. H., Franklandand, P. W., and Silva, A. J. (2000). Long-term memory underlying hippocampus-dependent social recognition in mice. *Hippocampus* 10, 47–56. doi:10.1002/(SICI)1098-1063(2000)10:1<47::AID-HIPO5>3.0.CO;2-6.

Kohl, C., Wang, X.-D., Grosse, J., Fournier, C., Harbich, D., Westerholz, S., et al. (2015). Hippocampal neuroligin-2 links early-life stress with impaired social recognition and increased aggression in adult mice. *Psychoneuroendocrinology* 55, 128–143. doi:10.1016/j.psyneuen.2015.02.016.

Koliatsos, V. E., Cernak, I., Xu, L., Song, Y., Savonenko, A., Crain, B. J., et al. (2011). A Mouse Model of Blast Injury to Brain: Initial Pathological, Neuropathological, and Behavioral Characterization. *J. Neuropathol. Exp. Neurol.* 70, 399–416. Available at: https://watermark.silverchair.com/70-5-399.pdf?token=AQECAHi208BE49Ooan9kkhW_Ercy7Dm3ZL_9Cf3qfKAc485ysgAAAaswggGnBgkqhkiG9w0BBwagggGYMIIBlAIBADCCAY0GCSqGSIb3DQEHATAeBglghkgBZQMEAS4wEQQM1TMSKLw0-jDeHw8sAgEQgIIBXvE8GgTev_fiy6umA86AigJ4xCi_TwTiRr7Kbx34vnMGlO [Accessed June 5, 2018].

Lan, W.-C. J., Priestley, M., Mayoral, S. R., Tian, L., Shamloo, M., and Penn, A. A. (2011). Sex-Specific Cognitive Deficits and Regional Brain Volume Loss in Mice Exposed to Chronic, Sublethal Hypoxia. *Pediatr. Res.* 70, 15–20. doi:10.1203/PDR.0b013e31821b98a3.

Lee, H.-J., Caldwell, H. K., Macbeth, A. H., Tolu, S. G., Young, W. S., and 3rd (2008). A conditional knockout mouse line of the oxytocin receptor. *Endocrinology* 149, 3256–63. doi:10.1210/en.2007-1710.

Lim, C. E., Turner, L. H., and Heinrichs, S. C. (2007). Short-term social recognition memory deficit and atypical social and physiological stressor reactivity in seizure-susceptible El mice. *Seizure* 16, 59–68. doi:10.1016/J.SEIZURE.2006.10.006.

Long, J. M., LaPorte, P., Paylor, R., and Wynshaw-Boris, A. (2004). Expanded characterization of the social interaction abnormalities in mice lacking Dvl1. *Genes. Brain. Behav.* 3, 51–62. Available at: http://www.ncbi.nlm.nih.gov/pubmed/14960015 [Accessed June 6, 2018].

Lukas, M., Toth, I., Veenema, A. H., and Neumann, I. D. (2013). Oxytocin mediates rodent social memory within the lateral septum and the medial amygdala depending on the relevance of the social stimulus: male juvenile versus female adult conspecifics. *Psychoneuroendocrinology* 38, 916–26. doi:10.1016/j.psyneuen.2012.09.018.

Macbeth, A. H., Edds, J. S., and Young, W. S. (2009). Housing conditions and stimulus females: a robust social discrimination task for studying male rodent social recognition. *Nat. Protoc.* 4, 1574–1581. doi:10.1038/nprot.2009.141.

Marino, M. D., Bourdélat-Parks, B. N., Cameron Liles, L., and Weinshenker, D. (2005). Genetic reduction of noradrenergic function alters social memory and reduces aggression in mice. *Behav. Brain Res.* 161, 197–203. doi:10.1016/j.bbr.2005.02.005.

McGowan, P. O., Hope, T. A., Meck, W. H., Kelsoe, G., and Williams, C. L. (2011). Impaired social recognition memory in recombination activating gene 1-deficient mice. *Brain Res.* 1383, 187–195. doi:10.1016/j.brainres.2011.02.054.

Moretti, P. (2006). Learning and Memory and Synaptic Plasticity Are Impaired in a Mouse Model of Rett Syndrome. *J. Neurosci.* 26, 319–327. doi:10.1523/JNEUROSCI.2623-05.2006.

Moura, P. J., Venkitaramani, D. V., Tashev, R., Lombroso, P. J., and Xavier, G. F. (2011). Transport of animals between rooms: A little-noted aspect of laboratory procedure that may interfere with memory. *Behav. Processes* 88, 12–19. doi:10.1016/j.beproc.2011.06.008.

Nelson, P. A., Sage, J. R., Wood, S. C., Davenport, C. M., Anagnostaras, S. G., and Boulanger, L. M. (2013). MHC class I immune proteins are critical for hippocampus-dependent memory and gate NMDAR-dependent hippocampal long-term depression. *Learn. Mem.* 20, 505–517. doi:10.1101/lm.031351.113.

Noack, J., Richter, K., Laube, G., Haghgoo, H. A., Veh, R. W., and Engelmann, M. (2010). Different importance of the volatile and non-volatile fractions of an olfactory signature for individual social recognition in rats versus mice and short-term versus long-term memory. *Neurobiol. Learn. Mem.* 94, 568–575. doi:10.1016/j.nlm.2010.09.013.

Nomoto, M., Takeda, Y., Uchida, S., Mitsuda, K., Enomoto, H., Saito, K., et al. (2012). Dysfunction of the RAR/RXR signaling pathway in the forebrain impairs hippocampal memory and synaptic plasticity. *Mol. Brain* 5, 8. doi:10.1186/1756-6606-5-8.

Perna, J. C., Wotjak, C. T., Stork, O., and Engelmann, M. (2015). Timing of presentation and nature of stimuli determine retroactive interference with social recognition memory in mice. *Physiol. Behav.* 143, 10–14. doi:10.1016/j.physbeh.2015.02.029.

Pierman, S., Sica, M., Allieri, F., Viglietti-Panzica, C., Panzica, G. C., and Bakker, J. (2008). Activational effects of estradiol and dihydrotestosterone on social recognition and the arginine-vasopressin immunoreactive system in male mice lacking a functional aromatase gene. *Horm. Behav.* 54, 98–106. doi:10.1016/j.yhbeh.2008.02.001.

Pietropaolo, S., Delage, P., Cayzac, S., Crusio, W. E., and Cho, Y. H. (2011). Sex-dependent changes in social behaviors in motor pre-symptomatic R6/1 mice. *PLoS One* 6, e19965. doi:10.1371/journal.pone.0019965.

Prediger, R. D. S., Rojas-Mayorquin, A. E., Aguiar, A. S., Chevarin, C., Mongeau, R., Hamon, M., et al. (2011). Mice with genetic deletion of the heparin-binding growth factor midkine exhibit early preclinical features of Parkinson’s disease. *J. Neural Transm.* 118, 1215–1225. doi:10.1007/s00702-010-0568-3.

Rial, D., Duarte, F. S., Xikota, J. C., Schmitz, A. E., Dafré, A. L., Figueiredo, C. P., et al. (2009a). Cellular prion protein modulates age-related behavioral and neurochemical alterations in mice. *Neuroscience* 164, 896–907. doi:10.1016/j.neuroscience.2009.09.005.

Rial, D., Xikota, J. C., Miozzo, A., Cruz, V. E. A., Prediger, R. D. S., and Walz, R. (2009b). Differential gender-related susceptibility to learning and memory deficits in mice submitted to neonatal freezing microgyria model. *Brain Res. Bull.* 79, 177–81. doi:10.1016/j.brainresbull.2009.02.003.

Richter, K., Wolf, G., and Engelmann, M. (2005). Social recognition memory requires two stages of protein synthesis in mice. *Learn. Mem.* 12, 407–13. doi:10.1101/lm.97505.

Sakamoto, M., Imayoshi, I., Ohtsuka, T., Yamaguchi, M., Mori, K., and Kageyama, R. (2011). Continuous neurogenesis in the adult forebrain is required for innate olfactory responses. *Proc. Natl. Acad. Sci. U. S. A.* 108, 8479–84. doi:10.1073/pnas.1018782108.

Samuelsen, C. L., and Meredith, M. (2011). Oxytocin antagonist disrupts male mouse medial amygdala response to chemical-communication signals. *Neuroscience* 180, 96–104. doi:10.1016/j.neuroscience.2011.02.030.

Sánchez-Andrade, G., and Kendrick, K. M. (2011). Roles of α- and β-estrogen receptors in mouse social recognition memory: Effects of gender and the estrous cycle. *Horm. Behav.* 59, 114–122. doi:10.1016/j.yhbeh.2010.10.016.

Satomoto, M., Satoh, Y., Terui, K., Miyao, H., Takishima, K., Ito, M., et al. (2009). Neonatal Exposure to Sevoflurane Induces Abnormal Social Behaviors and Deficits in Fear Conditioning in Mice. *Anesthesiology* 110, 628–637. doi:10.1097/ALN.0b013e3181974fa2.

Savonenko, A. V., Melnikova, T., Laird, F. M., Stewart, K.-A., Price, D. L., and Wong, P. C. (2008). Alteration of BACE1-dependent NRG1/ErbB4 signaling and schizophrenia-like phenotypes in BACE1-null mice. *Proc. Natl. Acad. Sci.* 105, 5585–5590. doi:10.1073/pnas.0710373105.

Scearce-Levie, K., Roberson, E. D., Gerstein, H., Cholfin, J. A., Mandiyan, V. S., Shah, N. M., et al. (2008). Abnormal social behaviors in mice lacking Fgf17. *Genes, Brain Behav.* 7, 344–354. doi:10.1111/j.1601-183X.2007.00357.x.

Schellinck, H. M., Rooney, E., and Brown, R. E. (1995). Odors of individuality of germfree mice are not discriminated by rats in a habituation-dishabituation procedure. *Physiol. Behav.* 57, 1005–8. Available at: http://www.ncbi.nlm.nih.gov/pubmed/7610125 [Accessed June 6, 2018].

Shiryaev, N., Pikman, R., Giladi, E., and Gozes, I. (2011). Protection against tauopathy by the drug candidates NAP (davunetide) and D-SAL: biochemical, cellular and behavioral aspects. *Curr. Pharm. Des.* 17, 2603–12. Available at: http://www.ncbi.nlm.nih.gov/pubmed/21728979 [Accessed June 6, 2018].

Sinai, L., Duffy, S., and Roder, J. C. (2010). Src inhibition reduces NR2B surface expression and synaptic plasticity in the amygdala. *Learn. Mem.* 17, 364–371. doi:10.1101/lm.1765710.

Steckler, T., Drinkenburg, W. H., Sahgal, A., and Aggleton, J. P. (1998). Recognition memory in rats--I. Concepts and classification. *Prog. Neurobiol.* 54, 289–311. Available at: http://www.ncbi.nlm.nih.gov/pubmed/9481800 [Accessed June 5, 2018].

Suzuki, A., Fukushima, H., Mukawa, T., Toyoda, H., Wu, L.-J., Zhao, M.-G., et al. (2011). Upregulation of CREB-mediated transcription enhances both short- and long-term memory. *J. Neurosci.* 31, 8786–802. doi:10.1523/JNEUROSCI.3257-10.2011.

Takayanagi, Y., Yoshida, M., Bielsky, I. F., Ross, H. E., Kawamata, M., Onaka, T., et al. (2005). Pervasive social deficits, but normal parturition, in oxytocin receptor-deficient mice. *Proc. Natl. Acad. Sci. U. S. A.* 102, 16096–101. doi:10.1073/pnas.0505312102.

Terranova, J. P., Pério, A., Worms, P., Le Fur, G., and Soubrié, P. (1994). Social olfactory recognition in rodents: deterioration with age, cerebral ischaemia and septal lesion. *Behav. Pharmacol.* 5, 90–98. Available at: http://www.ncbi.nlm.nih.gov/pubmed/11224255 [Accessed June 5, 2018].

Terranova, J. P., Storme, J. J., Lafon, N., Péŕio, A., Rinaldi-Carmona, M., Le Fur, G., et al. (1996). Improvement of memory in rodents by the selective CB1 cannabinoid receptor antagonist, SR 141716. *Psychopharmacology (Berl).* 126, 165–72. Available at: http://www.ncbi.nlm.nih.gov/pubmed/8856836 [Accessed June 5, 2018].

Thor, D. H., and Holloway, W. R. (1981). Persistence of social investigatory behavior in the male rat: Evidence for long-term memory of initial copulatory experience. *Anim. Learn. Behav.* 9, 561–565. Available at: https://link.springer.com/content/pdf/10.3758/BF03209791.pdf [Accessed June 5, 2018].

Thor, D. H., Wainwright, K. L., and Holloway, W. R. (1982). Persistence of attention to a novel conspecific: Some developmental variables in laboratory rats. *Dev. Psychobiol.* 15, 1–8. doi:10.1002/dev.420150102.

Van Loo, P. L. P., Van Zutphen, L. F. M., and Baumans, V. (2003). Male management: coping with aggression problems in male laboratory mice. *Lab. Anim.* 37, 300–313. doi:10.1258/002367703322389870.

Vulih-Shultzman, I., Pinhasov, A., Mandel, S., Grigoriadis, N., Touloumi, O., Pittel, Z., et al. (2007). Activity-Dependent Neuroprotective Protein Snippet NAP Reduces Tau Hyperphosphorylation and Enhances Learning in a Novel Transgenic Mouse Model. *J. Pharmacol. Exp. Ther.* 323, 438–449. doi:10.1124/jpet.107.129551.

Wang, Y., Zhao, S., and Liu, X. (2014). Effects of the medial or basolateral amygdala upon social anxiety and social recognition in mice. *Turkish J. Med. Sci.* 44, 353–359. doi:10.3906/sag-1301-2.

Wanisch, K., Wotjak, C., and Engelmann, M. (2008). Long-lasting second stage of recognition memory consolidation in mice. *Behav. Brain Res.* 186, 191–196. doi:10.1016/j.bbr.2007.08.008.

Wersinger, S. R., Caldwell, H. K., Martinez, L., Gold, P., Hu, S.-B., and Young, W. S. (2007). Vasopressin 1a receptor knockout mice have a subtle olfactory deficit but normal aggression. *Genes, Brain Behav.* 6, 540–551. doi:10.1111/j.1601-183X.2006.00281.x.

Wersinger, S. R., Ginns, E. I., O’Carroll, A.-M., Lolait, S. J., and Young III, W. S. (2002). Vasopressin V1b receptor knockout reduces aggressive behavior in male mice. *Mol. Psychiatry* 7, 975–984. doi:10.1038/sj.mp.4001195.

Winslow, J., and Insel, T. R. (2004). Neuroendocrine basis of social recognition. *Curr. Opin. Neurobiol.* 14, 248–253. doi:10.1016/j.conb.2004.03.009.

Winslow, J. T., and Camacho, F. (1995). Cholinergic modulation of a decrement in social investigation following repeated contacts between mice. *Psychopharmacology (Berl).* 121, 164–72. Available at: http://www.ncbi.nlm.nih.gov/pubmed/8545521 [Accessed June 6, 2018].

Xikota, J. C., Rial, D., Ruthes, D., Pereira, R., Figueiredo, C. P., Prediger, R. D. S., et al. (2008). Mild cognitive deficits associated to neocortical microgyria in mice with genetic deletion of cellular prion protein. *Brain Res.* 1241, 148–156. doi:10.1016/J.BRAINRES.2008.08.097.

Yang, L., Zou, B., Xiong, X., Pascual, C., Xie, J., Malik, A., et al. (2013). Hypocretin/orexin neurons contribute to hippocampus-dependent social memory and synaptic plasticity in mice. *J. Neurosci.* 33, 5275–84. doi:10.1523/JNEUROSCI.3200-12.2013.

Zhou, Z.-B., Yang, X.-Y., Yuan, B.-L., Niu, L.-J., Zhou, X., Huang, W.-Q., et al. (2015). Sevoflurane-Induced Down-regulation of Hippocampal Oxytocin and Arginine Vasopressin Impairs Juvenile Social Behavioral Abilities. *J. Mol. Neurosci.* 56, 70–77. doi:10.1007/s12031-014-0468-3.

Zou, H., Xie, Q., Zhang, M., Zhang, C., Zhao, G., Jin, M., et al. (2009). Chronic alcohol consumption from adolescence-to-adulthood in mice--effect on growth and social behavior. *Drug Alcohol Depend.* 104, 119–25. doi:10.1016/j.drugalcdep.2009.04.021.
